# Identification and validation of HOXC6 as a diagnostic biomarker for Ewing sarcoma: insights from machine learning algorithms and *in vitro* experiments

**DOI:** 10.3389/fimmu.2025.1449355

**Published:** 2025-04-04

**Authors:** Yonghua Pang, Jiahui Liang, Yakai Deng, Weinan Chen, Yunyan Shen, Jing Li, Xin Wang, Zhiyao Ren

**Affiliations:** ^1^ Department of Orthopedics, The 904th Hospital of the Joint Logistics Support Force, People's Liberation Army of China, Wuxi, Jiangsu, China; ^2^ Department of Breast Surgery, The First Affiliated Hospital of Anhui Medical University, Hefei, Anhui, China; ^3^ Department of General Surgery, The First Affiliated Hospital of Anhui Medical University, Hefei, Anhui, China; ^4^ Department of Orthopedics, Linyi People's Hospital, Linyi, Shandong, China; ^5^ Faculty of Medicine and Health Sciences, Ghent University, Ghent, Belgium

**Keywords:** Ewing sarcoma, bioinformatics, machine learning, HOXC6, diagnostic biomarker

## Abstract

**Introduction:**

Early diagnosis of Ewing sarcoma (ES) is critical for improving patient prognosis. However, the accurate diagnosis of ES remains challenging, underscoring the need for novel diagnostic biomarkers to enhance diagnostic precision and reliability. This study aimed to identify potential gene expression-based biomarkers for the diagnosis of ES.

**Methods:**

We selected the GSE17679, GSE45544, and GSE68776 datasets from the Gene Expression Omnibus (GEO) database. After correcting for batch effects, we combined ES and normal tissue samples from the GSE17679 and GSE45544 datasets to create a combined cohort. Two-thirds of both the tumor and normal samples from the combined cohort were randomly selected for the training cohort, while the remaining one-third served as the internal validation cohort. Additionally, the GSE68776 dataset was used for external validation. To identify key diagnostic genes, we applied three machine learning algorithms: least absolute shrinkage and selection operator (LASSO), support vector machine recursive feature elimination (SVM-RFE), and random forest (RF).

**Results:**

HOXC6 was identified as a key diagnostic biomarker for ES. It demonstrated strong diagnostic performance across all cohorts, with area under the curve (AUC) values of 0.956 (95% CI: 0.909−0.990) in the training cohort, 0.995 (95% CI: 0.977−1.000) in the internal validation cohort, and 0.966 (95% CI: 0.910−0.999) in the external validation cohort. Functional validation through HOXC6 knockdown in the RD-ES cell line revealed that its suppression significantly inhibited cell proliferation and migration. Furthermore, transcriptome sequencing suggested potential oncogenic mechanisms underlying HOXC6 function.

**Discussion:**

These findings highlight HOXC6 as a promising diagnostic biomarker for ES, demonstrating robust performance across multiple datasets. Additionally, its functional role suggests potential as a therapeutic target.

## 1 Introduction

Ewing sarcoma (ES) is an invasive malignant tumor that primarily affects bone and soft tissue. ES is a rare malignant tumor with an annual incidence rate not exceeding 1%. ES is most common in children and adolescents, typically between the ages of 10 and 20. The majority of cases involve male patients (1–3). ES is a complex disease driven by the coordination of multiple signaling pathways, with EWSR1/FLI1 identified as a key contributor to its pathogenesis (4, 5). The absence of precursor lesions makes the diagnosis and treatment of ES a challenge (6). ES is characterized by rapid growth and a tendency to undergo metastasis (7). The early diagnosis and treatment of ES can effectively prevent its recurrence and metastasis, thereby improving its prognosis (8). Currently, diagnostic methods for ES primarily rely on clinical symptoms, imaging, and pathology. However, achieving an accurate diagnosis remains relatively challenging. ES patients typically present with mild symptoms in the early stages and ES is easily confused with trauma, sports injuries, or growth-related discomfort, increasing the likelihood of delayed diagnosis and treatment (9). In addition, imaging techniques have significant limitations in diagnosing ES because of the lack of characteristic features and a high rate of misdiagnosis (10). Moreover, the pathological diagnosis of ES is complex, requiring molecular pathology and multiple diagnostic approaches to ensure accuracy, particularly in challenging cases (6). Therefore, there is an urgent need for a precise and simple diagnostic approach for the early detection of ES.

In recent years, with the completion of the Human Genome Project, high-throughput sequencing technology has undergone groundbreaking advancements. These developments have enabled the acquisition of more accurate gene expression profiles, the identification of disease-related genes, and an analysis of the mechanisms underlying complex diseases, driving the advent of the precision medicine era (11). Additionally, the emergence of machine learning, which allows computer systems to automatically learn from data and algorithms to improve their performance, has shown great potential in omics research (12). Collectively, these advancements offer considerable potential for the discovery of novel diagnostic biomarkers. To date, numerous studies have reported the successful use of machine learning techniques to identify diagnostic biomarkers for various tumors, such as lung cancer (13), colorectal cancer (14), and breast cancer (15). To our knowledge, no studies to date have focused on identifying diagnostic biomarkers for ES on the basis of transcriptome data. Therefore, this study aims to address this gap by identifying reliable diagnostic biomarkers derived from mRNA expression profiles.

**Figure 1:**
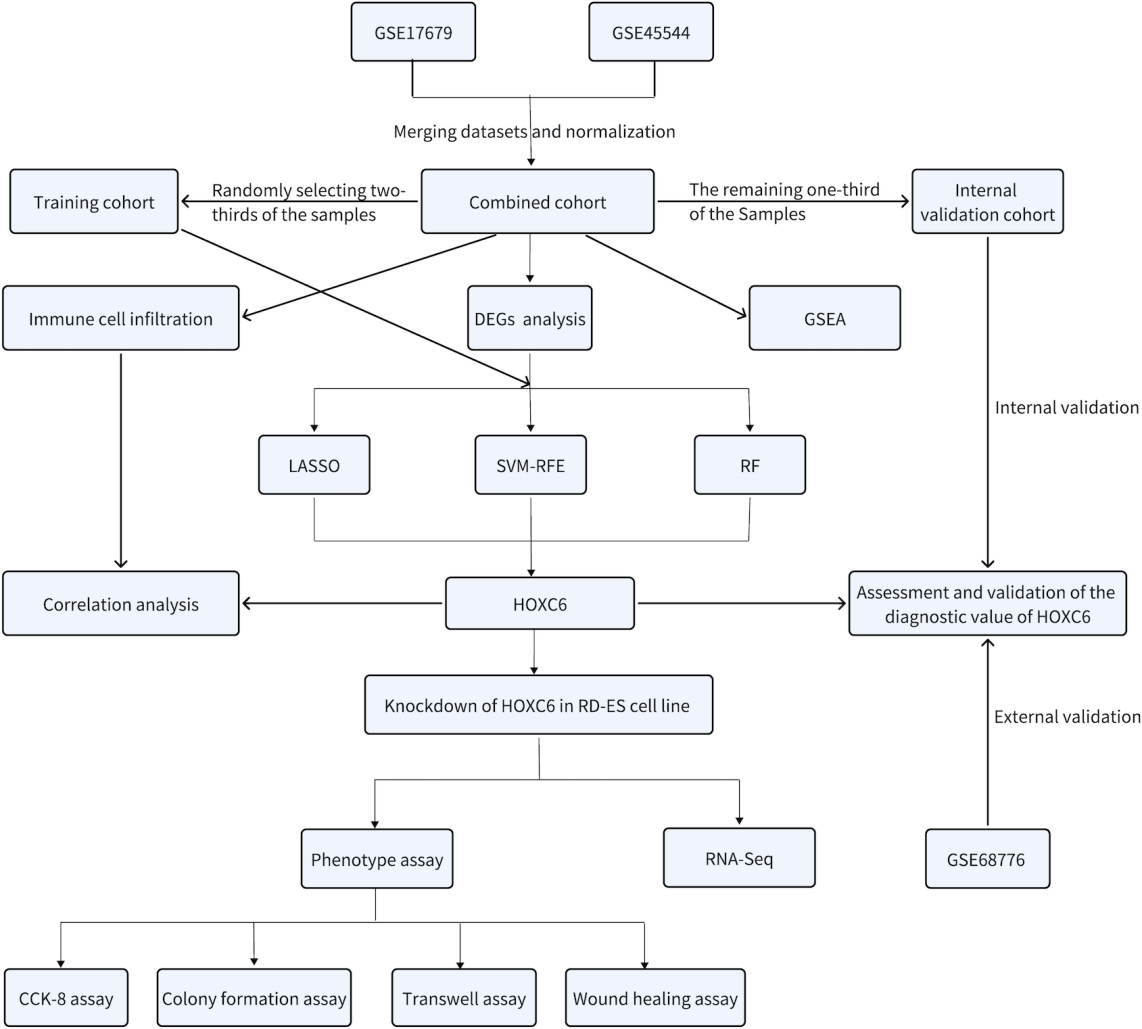
Flow chart of the comprehensive bioinformatics analysis and *in vitro* validation of HOXC6.

According to the flowchart shown in **Figure 1**, we first merged the GSE17679 and GSE45544 datasets using batch effect correction to create a combined cohort. From this cohort, two-thirds of both the tumor and normal tissue samples were randomly selected to form the training cohort, while the remaining one-third were designated as the internal validation. Additionally, the GSE68776 dataset was utilized for external validation. We performed gene set enrichment analysis (GSEA) on the combined cohort to explore functional and pathway enrichment differences between tumor and normal tissues. Next, we analyzed the gene expression profiles of ES and normal tissues within the combined cohort to identify differentially expressed genes (DEGs). Furthermore, on the basis of the DEGs identified, we used the training cohort to apply least absolute shrinkage and selection operator (LASSO), support vector machine recursive feature elimination (SVM-RFE), and random forest (RF) machine learning algorithms to identify diagnostic biomarkers associated with the pathogenesis of ES. Among these, HOXC6 emerged as the sole diagnostic biomarker. We subsequently evaluated and validated the diagnostic performance of HOXC6 using receiver operating characteristic (ROC) curve analysis in the training, internal, and external validation cohorts. Additionally, we assessed immune cell infiltration via the CIBERSORT algorithm and investigated the relationship between infiltrating immune cells and HOXC6 expression. Finally, we performed *in vitro* experiments in which HOXC6 was knocked down in the RD-ES cell line to explore its functional role.

## 2 Methods

### 2.1 Data collection and processing

We selected the GSE17679, GSE45544, and GSE68776 datasets from the Gene Expression Omnibus (GEO) database (**Table 1**). After removing batch effects using the surrogate variable analysis (SVA) algorithm (16), the ES and normal tissue samples from the GSE17679 and GSE45544 datasets were combined. We randomly selected two-thirds of the tumor tissues and two-thirds of the normal tissues from the combined cohort to construct the training cohort (68 tumor samples and 26 normal samples), while the remaining one-third was used for internal validation (34 tumor samples and 13 normal samples). We utilized the GSE68776 dataset for the purpose of external validation (32 tumor samples and 33 normal samples).

**Table 1:** Details of the datasets used in this study.

GEO series	Tissue sample		Cell line	
	ES	Normal	ES	Normal
GSE17679	88	18	0	0
GSE45544	14	21	7	1
GSE68776	32	33	0	0

### 2.2 Functional and biological pathway enrichment analyses

GSEA was conducted to identify significantly altered biological functions and signaling pathways between tumor and normal tissues in the combined cohort. For the purpose of this study, the Gene Ontology (GO), Kyoto Encyclopedia of Genes and Genomes (KEGG), and hallmark gene sets were downloaded from the Molecular Signatures Database (http://www.broadinstitute.org/msigdb) (17).

### 2.3 Identification of DEGs

The R package “limma” (18) was employed, applying the criteria of an absolute value of | log2(FC) |>1 and an FDR p value of <0.05 to identify DEGs between ES and normal tissues in the combined cohort. The DEGs were subsequently visualized using heatmaps and volcano plots.

### 2.4 Screening diagnostic biomarkers via three machine learning methods

In this study, LASSO (19), SVM-RFE (20) and RF (21) machine learning algorithms were independently applied to the training cohort to further screen diagnostic genes from the DEGs. The overlapping genes across the three sets of results were considered candidate diagnostic biomarkers for ES. LASSO logistic regression analysis was conducted using the “glmnet” package in R software. The SVM-RFE algorithm was applied using the “e1071” package in R software. The RF algorithm was implemented using the “randomForest” package in R software.

### 2.5 Assessment and validation of the diagnostic value of biomarkers in ES

To further assess the diagnostic value of the identified biomarkers in ES, ROC curves were generated, and the area under the curve (AUC) was calculated to evaluate and validate their predictive performance across the training, internal validation, and external validation cohorts. This analysis was performed using the R package “pROC”.

### 2.6 Evaluation of immune cell infiltration and correlation analysis between biomarkers and infiltrating immune cells

The quantification of different cell types involved in immune cell infiltration within ES gene expression profiles was conducted using the CIBERSORT algorithm (22). The correlations of infiltrating immune cells were visualized and analyzed using the R package “corrplot”. Additionally, a violin plot was generated using the R package “vioplot” to visualize the differences in infiltrating immune cells between ES and normal tissues. The correlation between diagnostic genes and immune cells was analyzed using the R packages “immuneCor” and “lollipop”. A lollipop chart was subsequently created to visualize the correlation between diagnostic gene levels and immune cell levels.

### 2.7 Cell culture

Human ES cells (RD-ES) were obtained from Qin Qi Biotechnology Development Co., Ltd., Shanghai, China. The cells were cultured in Dulbecco’s modified Eagle’s medium (DMEM) supplemented with 10% fetal bovine serum (FBS) under standard conditions in a humidified incubator with 5% CO_2_ at 37°C.

### 2.8 Cell transfection

To knockdown HOXC6 expression, specific short hairpin RNAs (shRNAs) were designed and transfected into RD-ES cells. The sequences of the shRNAs used were as follows: sh-HOXC6-1: 5′-TGCTGTTGACAGTGAGCGCGGAGACAGAAATAAATATTAATAGTGAAGCCACAGATGTATTAATATTTATTTCTGTCTCCATGCCTACTGCCTCGGA-3′; sh-HOXC6-2: 5′-TGCTGTTGACAGTGAGCGACAGTAGGAGAAAATAAATAAATAGTGAAGCCACAGATGTATTTATTTATTTTCTCCTACTGGTGCCTACTGCCTCGGA-3′. The knockdown efficiency was evaluated using real-time quantitative PCR (RT–qPCR) after 48 hours of transfection.

### 2.9 Real-time quantitative PCR

The sequences of the primers used in the experiment were as follows. For the HOXC6 gene, the primers used were as follows: forward primer, CCGTCAGTGTTCCTATCCAATTTTC; reverse primer, ATATTCGAGAACGGACCCAGAG. For ACTB, the primers used for the housekeeping gene were as follows: forward primer, CATGTACGTTGCTATCCAGGC; reverse primer, CTCCTTAATGTCACGCACGAT. After HOXC6 was knocked down in the RE-DS cell line, total mRNA was extracted from the cells with TRIzol reagent (TaKaRa, Japan). The concentration and purity were subsequently evaluated with a NanoDrop 2000 (Thermo Fisher, USA). The extracted RNA was then reverse transcribed into cDNA using the PrimeScript RT kit (TaKaRa, Japan) following the manufacturer’s instructions. RT–qPCR was subsequently performed using the SYBR Premix Ex Taq™ kit (TaKaRa, Japan) on an ABI StepOne Plus RT–qPCR system to detect SYBR Green fluorescence signals after each amplification cycle. Data processing was performed using GraphPad Prism 10.0.0, and a t test was conducted to compare the values for the experimental group with those of the control group.

### 2.10 Cell proliferation assay

Proliferation assays were performed over five consecutive days on cells seeded in a 96-well plate using the Cell Counting Kit-8 (CCK-8) reagent (Beyotime, China). A total of 2000 cells were plated per well and incubated at 37°C. The absorbance at 450 nm was measured daily for five days using a microplate reader.

### 2.11 Colony formation assays

Approximately 2000 cells per well were seeded into a 6-well culture plate and incubated at 37°C for two weeks. After being washed with PBS twice, the cells were fixed with 4% paraformaldehyde for 15 min and then stained with crystal violet. Each experiment was repeated three times. ImageJ was used for image analysis to convert images into cellular count data (23). The acquired counts were normalized by dividing them by the corresponding cell count in the control group, yielding percentage data. Data and image processing were performed using GraphPad Prism 10.0.0 and ImageJ. The statistical analysis consisted of a t test conducted on three replicate datasets to compare the values between the experimental and control groups.

### 2.12 Migration assays

A 24-well Transwell plate (Costar) was used for the cell migration assays. First, 50 μl of serum-free Ham’s F-12K medium was added to the upper chamber of the Transwell plate without the addition of a matrix. The plate was incubated at 37°C for 30 minutes. The knockdown cells or negative control (NC) cells were prepared as described previously. After a 20-minute incubation, 100 μl of the shRNA mixture was mixed with 100 μl of serum-free Ham’s F-12K containing 1×10^5^ cells. The mixture was transferred to the upper chamber of the Transwell system. In the lower chamber, 500 μl of Ham’s F-12K medium supplemented with 10% FBS was added. The mixture was incubated at 37°C for 24 hours. A cotton swab was used to remove nonmigrated cells from the lower chamber. The upper chamber was removed, and the cells were washed with PBS. The cells were fixed and stained with Giemsa. Five fields were randomly selected under an optical microscope, and the migrated cells were counted.

### 2.13 Wound healing assay

Wound healing assays were performed following previously described protocols (24). Briefly, cells were seeded in 6-well plates and incubated at 37°C until reaching full confluence. A scratch was then made across the middle of each well to create a wound, and the medium was replaced with serum-free medium. After 48 hours, the wound area was measured.

### 2.14 RNA-seq

RD-ES cells were subjected to RNA sequencing after HOXC6 knockdown. Approximately 2 μg of total RNA was extracted from each sample and pretreated with the Epicenter Ribo-zero™ rRNA Removal Kit. An RNA library was then constructed following the manufacturer’s protocol for the NEBNext^®^ Ultra™ Directional RNA Library Prep Kit (NEB, USA). The procedure was as follows: RNA was first fragmented into small pieces by treatment with NEBNext First Strand Synthesis Reaction Buffer at high temperature, and first-strand cDNA was synthesized using random hexamer primers and M-MuLV reverse transcriptase. Next, second-strand cDNA was synthesized, and the fragment ends were repaired to blunt ends using exonuclease or polymerase. The 3’ ends of the cDNA fragments were then adenylated and ligated to NEBNext adapters with a hairpin structure. After purification with the AMPure XP system (Beckman Coulter, Beverly, USA), 150–200 bp DNA fragments were selected and sequenced using the HiSeq 2500 platform (Illumina, CA, USA).

### 2.15 RNA-seq data processing and analysis

The FastQC program (http://www.bioinformatics.babraham.ac.uk/projects/fastqc/) was used to assess the sequencing quality of all the sample data, which were trimmed using the FASTX-Toolkit. The sequencing reads were mapped to the human reference genome assembly GRCh37 using TopHat (v2.0.9). Differential expression analysis was conducted on the basis of the gene expression matrix in count format. The R package edgeR was used to compare differential gene expression between shHOXC6-expressing cells and the NC group using a predetermined threshold (q value > 0.05). Volcano plots were generated to visualize the differentially expressed genes. Intersection analyses were performed separately for the upregulated and downregulated genes to identify the genes that were consistently differentially expressed across both replicates. GO and KEGG enrichment analyses were subsequently performed on the upregulated and downregulated genes, with a significance threshold of a q value < 0.05.

## 3 Results

### 3.1 Functional and biological pathway enrichment analyses

After correction, we merged the tissue samples from the GSE17679 and GSE45544 datasets, and the merging effect was satisfactory, allowing us to construct a combined cohort (**Supplementary Figures 1A, B**). To explore the biological pathways associated with ES, we performed GSEA using the cancer GO, KEGG, and hallmark gene sets. The results demonstrated that ES tissues were enriched in pathways associated with chromosomal abnormalities, the cell cycle, ribosomes, and the epithelial–mesenchymal transition (EMT) (**Supplementary Figure 2**, **Supplementary Tables 1**–**3**).

### 3.2 Identification of DEGs and diagnostic biomarker screening using three machine learning methods

**Figure 2:**
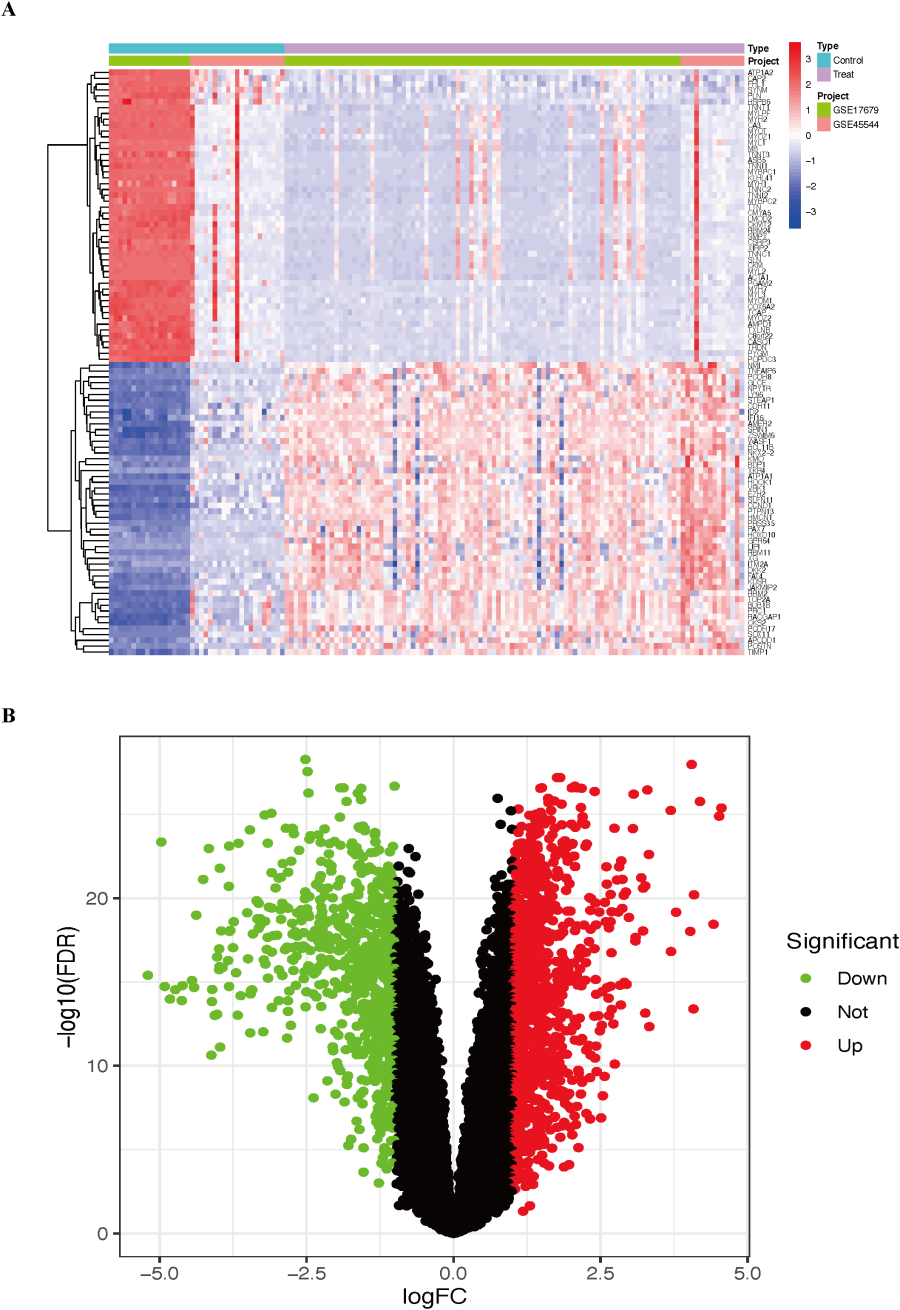
The results include the top 50 upregulated and downregulated DEGs identified in the combined cohort. **(A)** Heatmap of the DEGs. **(B)** Volcano plot of the DEGs. ES, Ewing sarcoma; DEGs, differentially expressed genes.

DEGs were identified in the combined cohort. We identified a total of 1832 DEGs, comprising 1077 upregulated genes and 755 downregulated genes (**Supplementary Table 4**). DEGs were visualized using heatmaps (**Figure 2A**) and volcano plots (**Figure 2B**).

**Figure 3:**
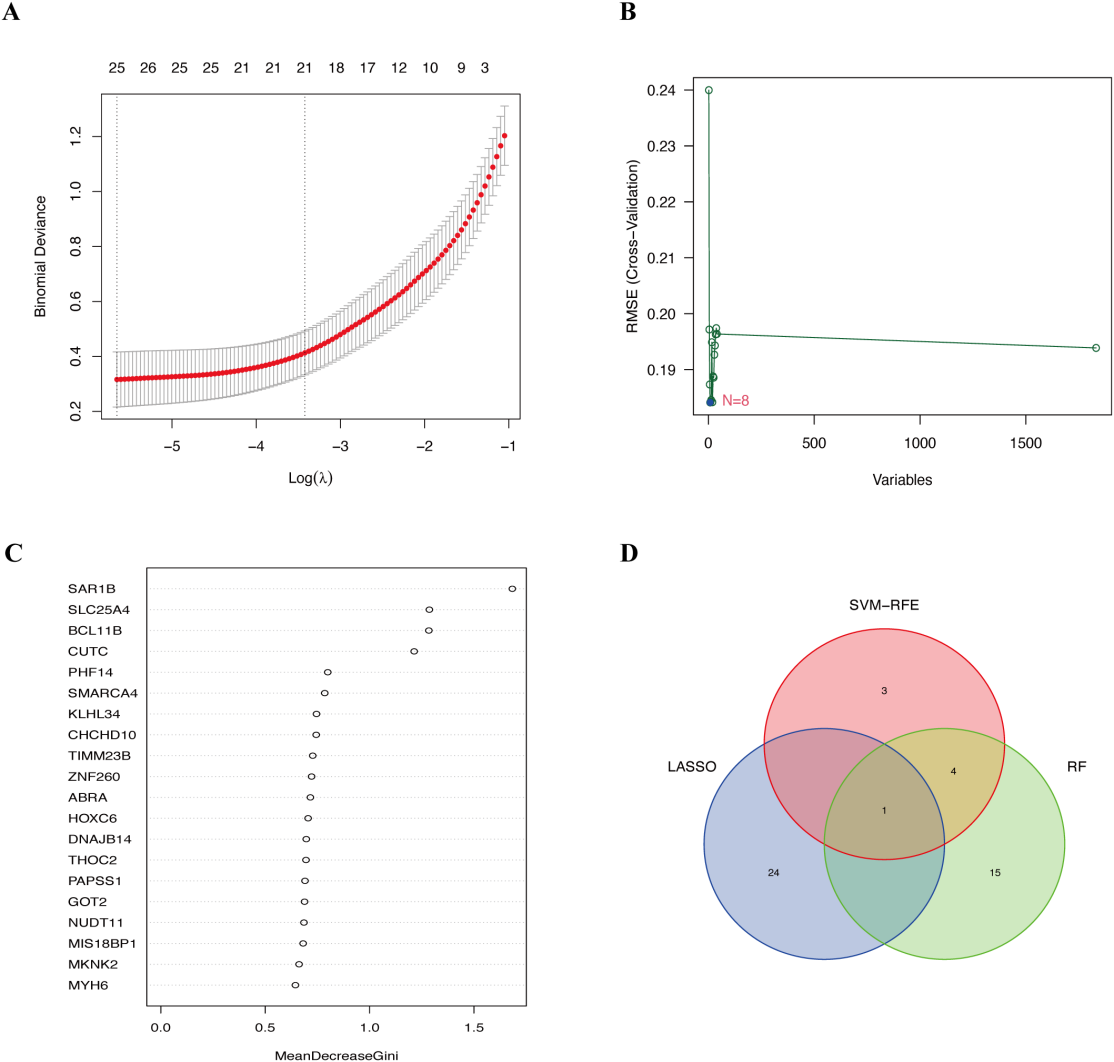
Diagnostic biomarkers screened via three machine learning algorithms. **(A)** Identification of diagnostic biomarkers by LASSO regression analysis. **(B)** Selection of diagnostic biomarkers using the SVM-RFE algorithm. **(C)** Detection of diagnostic biomarkers through the RF algorithm. **(D)** Venn diagram showing the overlapping biomarkers across the LASSO, SVM-RFE and RF analyses. LASSO, least absolute shrinkage and selection operator; SVM-RFE, support vector machine recursive feature elimination; RF, random forest.

On the basis of the DEGs, we further employed three machine learning algorithms—LASSO, SVM-RFE, and RF—to screen for diagnostic biomarkers of ES in the training group, identifying 25, 8, and 20 potential diagnostic genes, respectively (**Figures 3A–C**). HOXC6 was identified as the only overlapping gene across the three machine learning analyses (**Figure 3D**). HOXC6 expression is significantly higher in tumor tissues than in normal tissues (**Supplementary Figure 3**). Interestingly, we also found that HOXC6 expression levels were higher in tumor cell lines than in the normal cell line on the basis of the GSE45544 dataset (**Supplementary Figure 4**). Furthermore, HOXC6 expression was elevated in multiple tumor types, including stomach adenocarcinoma, invasive breast carcinoma, and esophageal carcinoma (**Supplementary Figure 5**), highlighting its critical role in various cancers.

### 3.3 Assessment and validation of the diagnostic value of HOXC6 in ES

**Figure 4:**
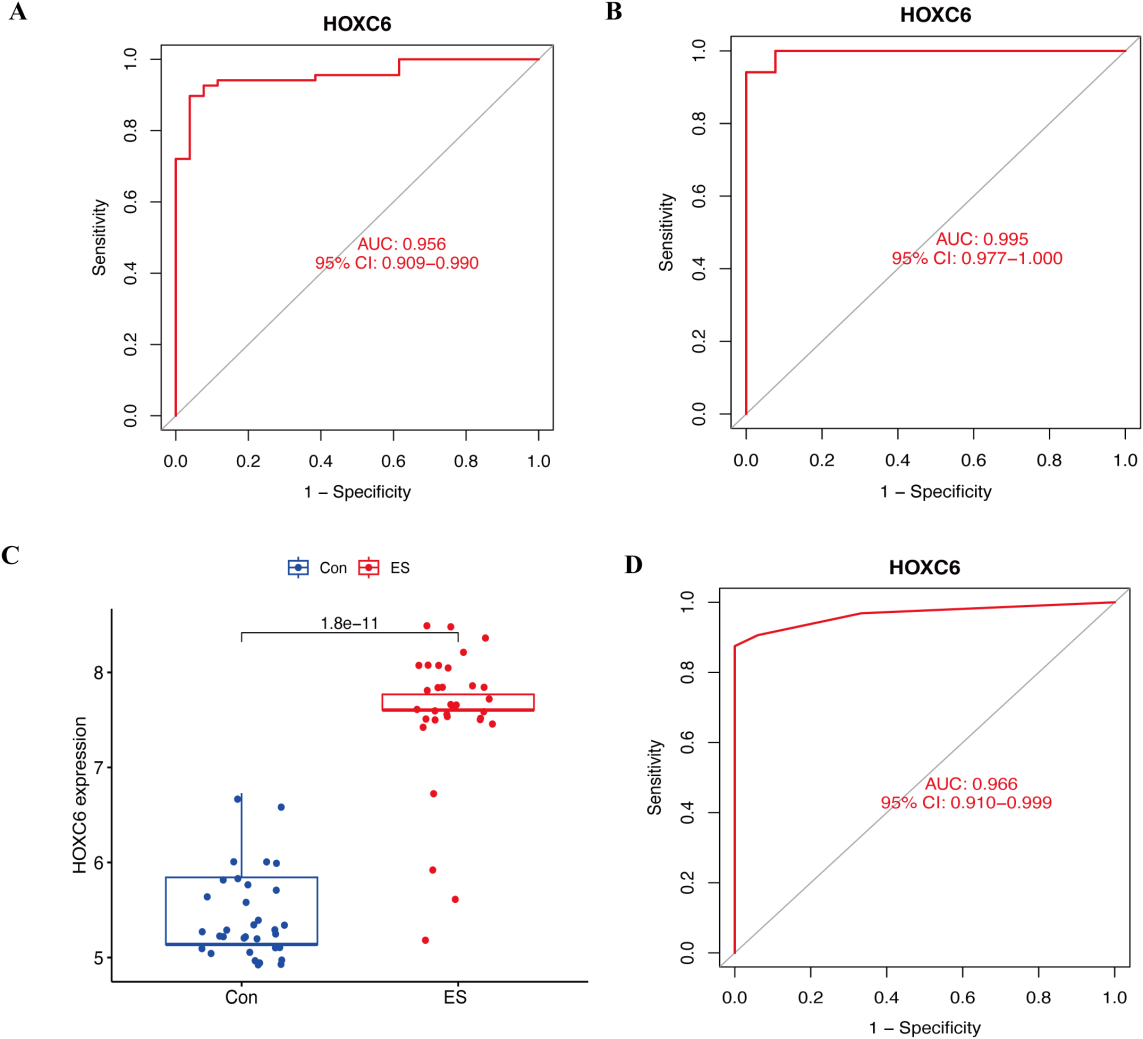
Assessment and validation of the diagnostic value of HOXC6 in ES. **(A)** ROC curve for assessing the diagnostic efficacy of HOXC6 in the training cohort. **(B)** ROC curve for validating the diagnostic efficacy of HOXC6 in the internal validation cohort. **(C)** Box plots of the expression of HOXC6 in ES and normal tissues in the external validation cohort. **(D)** ROC curve for validating the diagnostic efficacy of HOXC6 in the external validation cohort. ES, Ewing sarcoma.

To further assess and validate the diagnostic value of HOXC6 in ES, ROC analysis was conducted for HOXC6 across the training, internal, and external validation cohorts. The results demonstrated that HOXC6 exhibited strong diagnostic performance in the training cohort, with an AUC of 0.956 (95% CI: 0.909−0.990) (**Figure 4A**). Similarly, in the internal validation cohort, HOXC6 showed excellent diagnostic accuracy, with an AUC of 0.995 (95% CI: 0.977−1.000) (**Figure 4B**). Furthermore, HOXC6 was highly expressed in tumor tissues in the external validation cohort (**Figure 4C**) and maintained strong diagnostic efficacy, with an AUC of 0.966 (95% CI: 0.910−0.999) (**Figure 4D**). These results indicate that HOXC6 is a reliable diagnostic biomarker for ES.

### 3.4 Evaluation of immune cell infiltration and correlation analysis between HOXC6 and infiltrating immune cells

**Figure 5:**
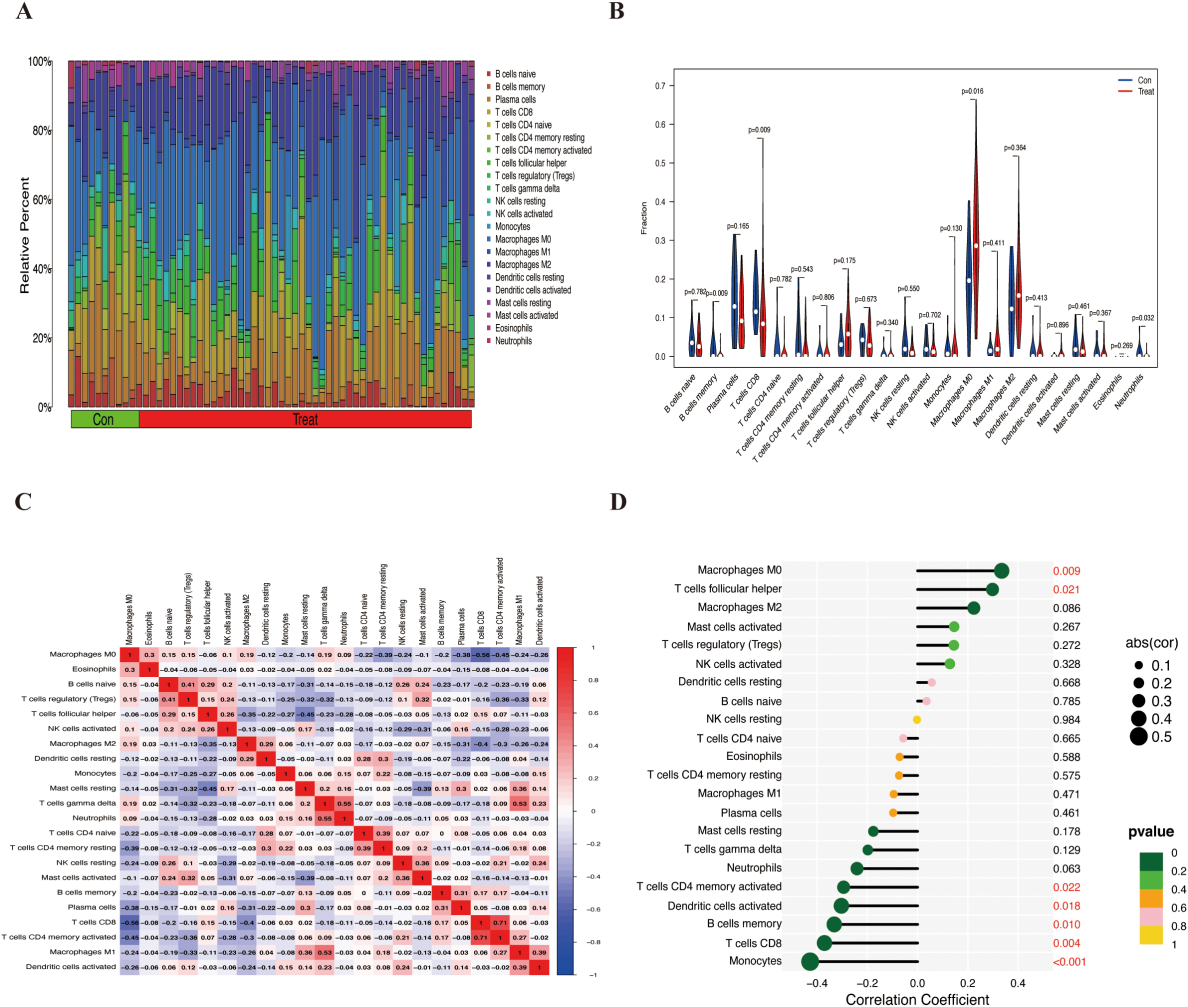
The infiltration of immune cells in the combined cohort and its correlation with HOXC6. **(A)** Bar chart of the proportions of 22 types of infiltrating immune cells. **(B)** Differential analysis of 22 types of infiltrating immune cells. **(C)** Heatmap showing the correlations between 22 immune cells. **(D)** Correlations between HOXC6 and 22 types of immune cells.

Initially, we assessed the infiltration of immune cells in the combined cohort using the CIBERSORT algorithm (**Figure 5A**). Compared with that in normal tissues, the degree of M0 macrophage infiltration in ES tissues was significantly greater (*p*=0.016). Conversely, the degree of infiltration of memory B cells (*p*=0.009) and CD8+ T cells (*p*=0.009) was greater in normal tissues than in ES tissues (**Figure 5B**). Furthermore, we calculated the correlations between the 22 types of infiltrating immune cells (**Figure 5C**).

HOXC6 expression was significantly positively correlated with the number of M0 macrophages (*p* = 0.009) and follicular helper T cells (*p* = 0.021) and significantly negatively correlated with the number of CD4 memory-activated T cells (*p* = 0.022), activated dendritic cells (*p* = 0.018), memory B cells (*p* = 0.010), CD8+ T cells (*p* = 0.004), and monocytes (*p* < 0.001) (**Figure 5D**). These findings suggest that HOXC6 may play a role in the tumor immune microenvironment.

### 3.5 Knockdown of HOXC6 inhibited ES cell proliferation and migration

**Figure 6:**
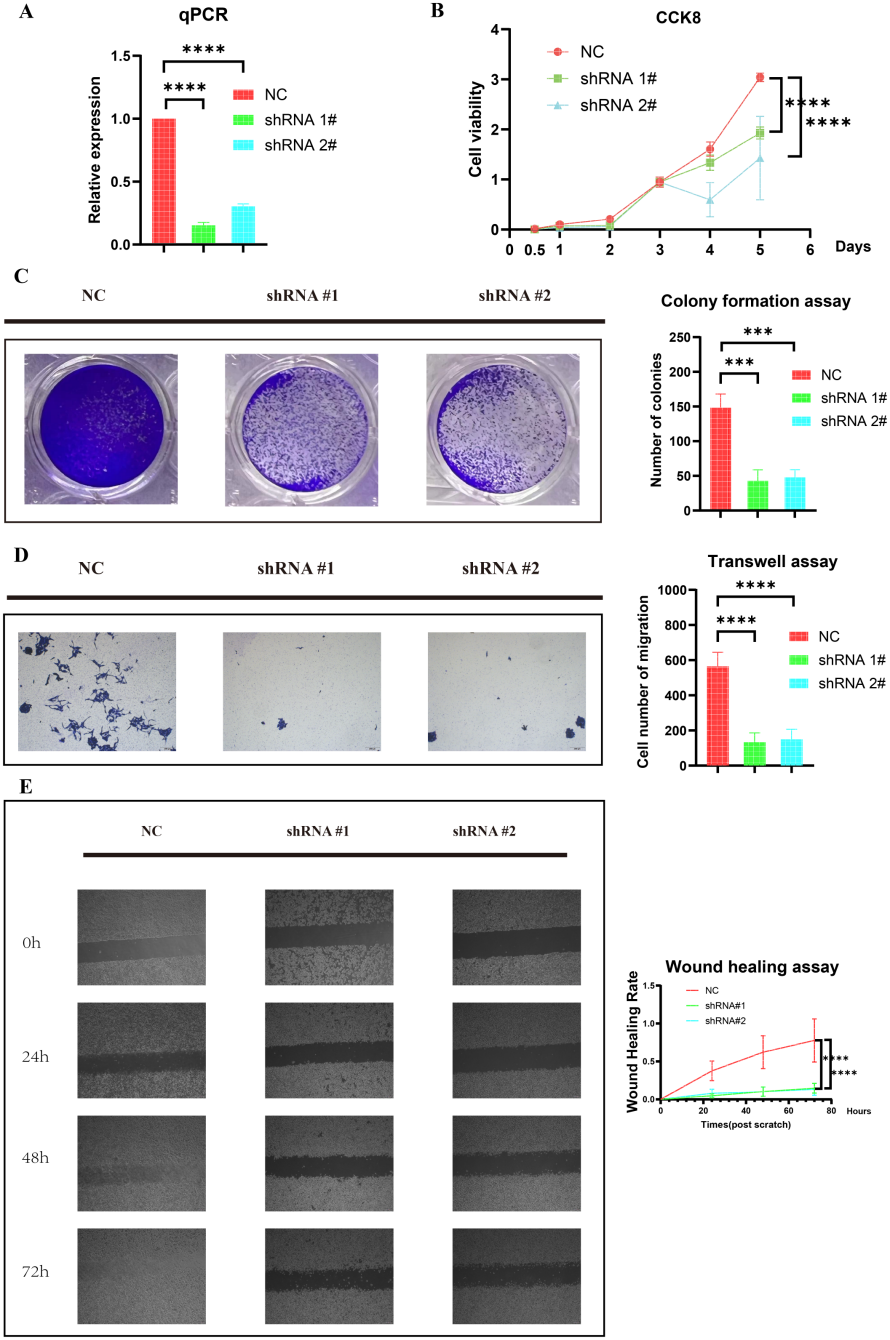
Knockdown of HOXC6 inhibited ES cell proliferation and migration. **(A)** Knockdown efficiency of HOXC6 in the RD-ES shHOXC6 cell line. **(B)** CCK-8 assay. **(C)** Colony formation assay. **(D)** Transwell assay. **(E)** Wound healing assay. ES, Ewing sarcoma. The data is presented as the mean from at least three independent experiments. (***p< 0.001; ****p< 0.0001).

We established two RD-ES shHOXC6 cell lines through lentiviral transduction. As shown in **Figure 6A**, the HOXC6 knockdown efficiency in RD-ES cells was satisfactory.

We further investigated the impact of HOXC6 knockdown on the proliferation of ES cells through CCK-8 and colony formation assays. As shown in **Figure 6B**, the CCK-8 assay revealed that HOXC6 knockdown suppressed RD-ES cell proliferation. Similarly, the colony formation assay confirmed a significant reduction in proliferation (**Figure 6C**). Together, these results suggest that HOXC6 positively regulates ES cell proliferation.

We next examined the cell migration ability. Transwell assays demonstrated that HOXC6 knockdown significantly inhibited the migration of RD-ES cells (**Figure 6D**). Similarly, the wound healing assay results revealed that reduced HOXC6 expression impaired the wound closure rate of RD-ES cells (**Figure 6E**). These findings suggest that HOXC6 functions as an oncogene in the progression of ES.

### 3.6 Transcriptome-based analysis of HOXC6-related pathways

**Figure 7:**
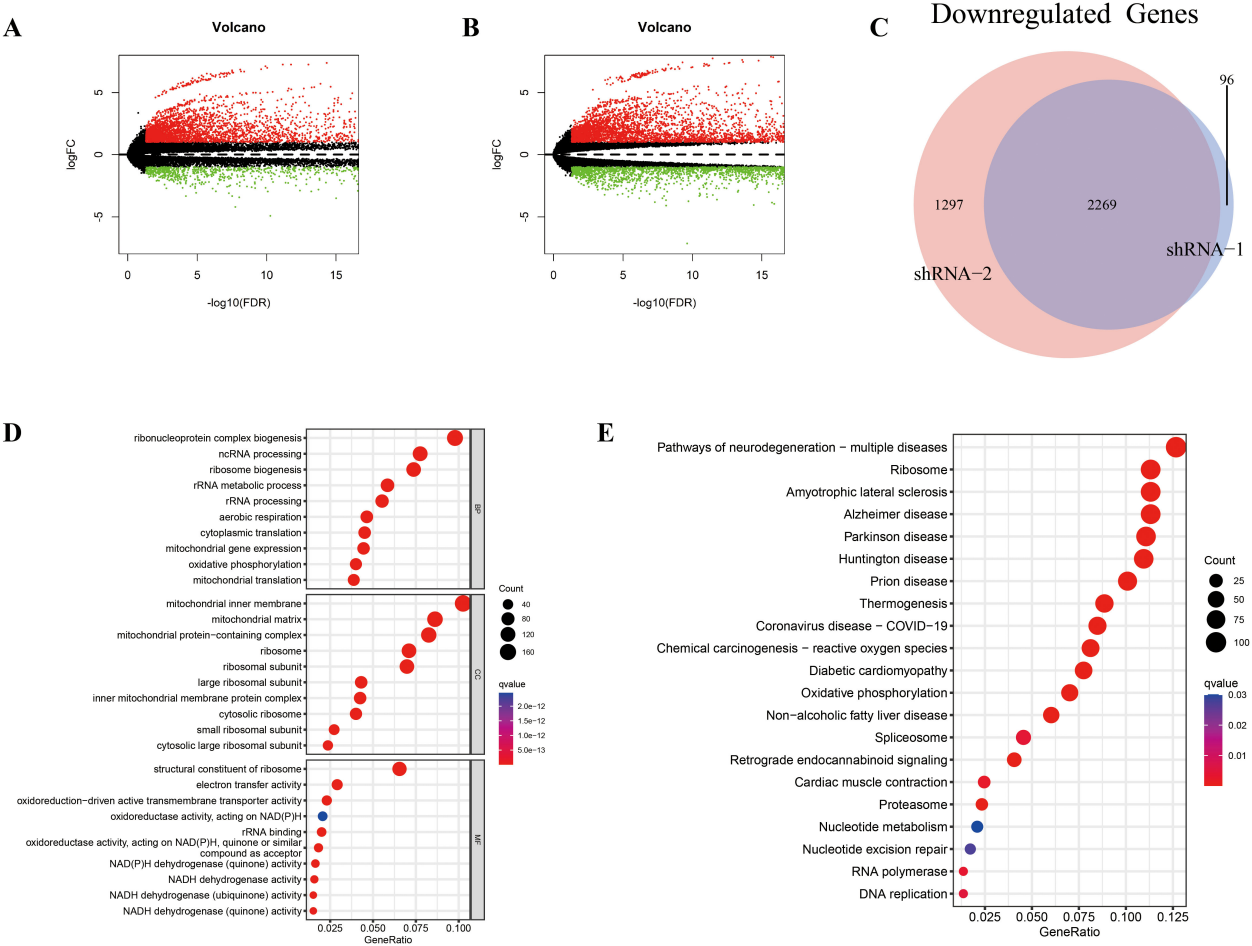
Differential expression analysis and enrichment analysis between the shHOXC6 and control cell lines. **(A-E)** Volcano plot displaying the differentially expressed genes between the shHOXC6_1 **(A)** and shHOXC6_2 **(B)** groups and the NC group. **(C)** Venn diagram showing the overlap in downregulated genes between the shHOXC6_1 and shHOXC6_2 groups compared with the NC group; GO **(D)** and KEGG **(E)** enrichment analysis of the overlapping downregulated genes. NC, negative control.

We performed transcriptome sequencing on two RD-ES shHOXC6 cell lines and the NC group, analyzing all upregulated and downregulated genes between the two knockdown cell lines and the NC group (**Figures 7A, B**, **Supplementary Tables 5**, **6**). First, we identified the intersection of commonly downregulated genes (**Figure 7C**, **Supplementary Table 7**) and conducted GO and KEGG analyses (**Figures 7D, E**, **Supplementary Tables 8**, **9**). The results revealed associations with ribosomes, metabolism, and the cell cycle. Functional enrichment analysis was also performed on the intersecting upregulated genes (**Supplementary Figure 6**, **Supplementary Tables 10**−**12**).

## 4 Discussion

ES ranks as the second most prevalent malignant bone tumor among children and adolescents (25). Although significant progress has been made in the early diagnosis and treatment of ES in recent years, the absence of specific diagnostic biomarkers still presents significant challenges for achieving accurate early diagnosis of ES. Reports in the literature indicate that up to 25% of patients present with metastasis at the time of diagnosis, contributing to a persistently poor clinical prognosis (26). Thus, the identification of diagnostic biomarkers for ES is crucial for improving the diagnostic accuracy and prognosis of ES. To our knowledge, this study is among the first to explore diagnostic biomarkers for ES on the basis of transcriptome data.

The GSEA comparing ES and normal tissues in this study revealed that chromosomal abnormalities, the cell cycle, ribosomes, and the EMT may play critical roles in the development and progression of ES. Chromosomal translocation involving EWSR1/FLI1 is a key driver in the pathogenesis of ES (27). While the EMT is essential for normal embryonic development and tissue regeneration, its aberrant reactivation is associated with tumor malignancy, contributing to cancer progression and metastasis (28).

As genomic technology advances, there is a growing trend toward the use of bioinformatics analysis methods to explore the molecular mechanisms underlying gene expression profiles (29). This approach holds great promise for identifying specific molecular diagnostic markers or therapeutic targets for various diseases (30). LASSO analysis is a regression method known for its ability to efficiently process large datasets, effectively perform parameter shrinkage and variable selection, prevent overfitting, and more accurately screen variables (31). In omics research, the screening of differentially expressed biomarkers from tissue data is pivotal. SVM-RFE has emerged as an efficient technique for feature selection and has promising application prospects in the analysis of metabolomics data (20). RF is a flexible and powerful machine learning algorithm that offers advantages such as high accuracy, resistance to overfitting, the ability to handle missing data, and built-in feature selection. It is particularly well suited for complex datasets and high-dimensional problems (21). To identify reliable diagnostic biomarkers, we first performed differential gene expression analysis in the combined cohort. On the basis of the identified DEGs, we applied three machine learning methods in the training cohort and identified HOXC6 as the only diagnostic biomarker through intersection analysis. ROC curves demonstrated the excellent predictive performance of HOXC6 for ES. Internal validation is crucial for estimating the generalizability of a model (32). In this study, the strong results from internal validation provide robust evidence supporting the reliability of the diagnostic biomarker HOXC6. Furthermore, external validation is essential for assessing the reproducibility of the model and its applicability to independent samples (33). The GSE68776 dataset was used for external validation in this study. The AUCs obtained in this study were satisfactory, indicating the reproducibility of HOXC6 use for ES diagnosis in practical applications.

HOXC6 plays a pivotal role in regulating embryonic development, cell differentiation, and organ formation (34). As one of the 39 HOX genes in humans (35), HOXC6 is overexpressed in several cancers, including osteosarcoma (36), lung adenocarcinoma (37), and prostate cancer (38). It is critically involved in tumor cell proliferation, growth, and metastasis by regulating various proteins, such as bone morphogenetic protein 7 (BMP7) (39, 40). Liu et al. (41) reported that HOXC6 is involved in various processes, including immune cell infiltration, immune-related genes, chemotherapy sensitivity, signaling pathways, and transcriptional regulatory networks. Moreover, it may function as a radiosensitivity-related gene affecting the prognosis of rectal cancer patients and could serve as a potential target for radiotherapy. Huang et al. (42) noted that HOXC6 may play a significant role in promoting tumor development and glioma progression by regulating the EMT signaling pathway. Additionally, it may serve as a novel immunotherapeutic target for glioma treatment. Wang et al. (43) reported that HOXC6 overexpression enhances BCL2-mediated antiapoptotic effects, thereby promoting cervical cancer cell cycle progression and proliferation. These studies indicate that HOXC6 plays an important role in tumor diseases.

The tumor microenvironment plays a crucial role in all stages of cancer progression (44). In this study, we observed that CD8+ T-cell infiltration was significantly lower in ES tissues than in normal tissues and was negatively correlated with HOXC6 expression. CD8+ T cells, also known as cytotoxic T cells, can recognize and directly kill tumor cells. However, in the tumor immune microenvironment, CD8+ T cells often experience exhaustion (45). Previous studies have indicated that HOXC6 can regulate the tumor immune microenvironment (42). Hence, our results indicate that HOXC6 may promote ES progression by mediating the exhaustion of CD8+ T cells.

To investigate the mechanisms by which HOXC6 promotes tumor progression, we performed transcriptome sequencing. Differential expression analysis between the knockdown and NC groups, with a focus on downregulated genes, revealed significant enrichment of ribosome-related pathways in both the GO and KEGG analyses. Interestingly, ribosome-related pathways were also enriched in the GSEA between ES and normal tissues. The ribosome, a complex molecular machine responsible for protein synthesis, has been shown to play critical roles in tumor proliferation, growth, and metastasis (46, 47). To date, no studies have reported that HOXC6 mediates tumor progression by regulating ribosomes. Therefore, this study may reveal a novel oncogenic mechanism of HOXC6 and highlight its potential as a therapeutic target.

In the future, the collection of clinical samples will be essential for validating the diagnostic accuracy of our findings. Additionally, further investigations utilizing a wider array of *in vitro* models, including diverse cell lines and patient-derived primary cells, as well as *in vivo* studies, will offer valuable insights into the role of HOXC6 in the pathogenesis of ES. Moreover, transcriptomic data can be leveraged to elucidate the downstream mechanisms regulated by HOXC6. Further experiments are needed to elucidate the downstream mechanisms of HOXC6. Overall, this study provides a simple and efficient diagnostic strategy for ES based on the expression of a single gene, HOXC6, which holds great potential for clinical application. Additionally, our findings establish HOXC6 as an oncogene, highlighting its promise as a therapeutic target pending further comprehensive research.

## 5 Conclusion

In this study, a simple and efficient diagnostic strategy for ES was developed on the basis of the expression of a single gene, HOXC6, which holds great potential for clinical application. Additionally, *in vitro* experiments revealed HOXC6 as an oncogene in ES tumorigenesis, highlighting its promise as a therapeutic target pending further comprehensive research.

## Data Availability

The data presented in this study are deposited in the GEO database repository, accession number GSE263430.

## Supplementary material

Supplementary Table 1 GSEA of ES and normal tissues based on GO biological processes. GSEA, gene set enrichment analysis; ES, Ewing sarcoma; GO, Gene Ontology.

Supplementary Table 2 GSEA of ES and normal tissues based on the KEGG pathway analysis. GSEA, gene set enrichment analysis; ES, Ewing sarcoma; KEGG, Kyoto Encyclopedia of Genes and Genomes.

Supplementary Table 3 GSEA of ES and normal tissues on the basis of hallmark pathways. GSEA, gene set enrichment analysis; ES, Ewing sarcoma.

Supplementary Table 4 Differentially expressed genes between ES and normal tissues in the combined cohort. ES, Ewing sarcoma.

Supplementary Table 5 Differentially expressed genes between the shHOXC6_1 and NC groups.

Supplementary Table 6 Differentially expressed genes between the shHOXC6_2 and NC groups.

Supplementary Table 7 Downregulated gene sets.

Supplementary Table 8 GO enrichment analysis of the downregulated gene sets. GO, Gene Ontology.

Supplementary Table 9 KEGG enrichment analysis of the downregulated gene sets. KEGG, Kyoto Encyclopedia of Genes and Genomes.

Supplementary Table 10 Upregulated gene sets.

Supplementary Table 11 GO analysis of the upregulated gene sets. GO, Gene Ontology.

Supplementary Table 12 KEGG pathway analysis of the upregulated gene sets. KEGG, Kyoto Encyclopedia of Genes and Genomes.
