# Supplementary material for: Identification and validation of HOXC6 as a diagnostic biomarker for Ewing sarcoma: insights from machine learning algorithms and in vitro experiments
Source: Front Immunol. 2025 Apr 4;16:1449355. doi: 10.3389/fimmu.2025.1449355 (PMC12006176; doi:10.3389/fimmu.2025.1449355)
Supplement: Supplementary Table 1 — GSEA of ES and normal tissues based on GO biological processes. GSEA, gene set enrichment analysis; ES, Ewing sarcoma; GO, Gene Ontology. [file DataSheet2.zip › Supplementary material presentation/Supplementary Material.docx]

Supplementary Material

# Supplementary Figures and Tables

## Supplementary Figures

**Supplementary Figure 1.** The batch correction of the combined cohort. **(A)** The boxplots of the combined cohort before and after batch correction. **(B)** The PCA analysis of the combined cohort before and after batch correction.

**Supplementary Figure 2.** GSEA of ES and normal tissues. **(A, B)** GSEA-GO analysis. **(C, D)** GSEA-KEGG analysis. **(E, F)** GSEA-hallmark analysis. GSEA, Gene Set Enrichment Analysis; ES, Ewing sarcoma; GO, Gene Ontology; KEGG, Kyoto Encyclopedia of Genes and Genomes.

**Supplementary Figure 3.** Box plots of the expression of HOXC6 between ES and normal tissues in the combined cohort. ES, Ewing sarcoma.

**Supplementary Figure 4.** Box plots of the expression of HOXC6 between ES and normal cell line samples in the GSE45544 dataset.

**Supplementary Figure 5.** TIMER 2.0 analysis indicated that SENP5 is highly expressed in most tumor types.

**Supplementary Figure 6.** Differential analysis and enrichment analysis between shHOXC6 and control cell lines. **(A)** The Venn diagram shows the overlap in upregulated expressed genes between shHOXC6_1 and shHOXC6_2 compared to the NC group; GO **(B)** and KEGG **(C)** enrichment analysis of the overlapping upregulated expressed genes. GO, Gene Ontology; KEGG, Kyoto Encyclopedia of Genes and Genomes.

## Supplementary Tables

**Supplementary Table 1.** GSEA of ES and normal tissues based on GO biological processes.

**Supplementary Table 2.** GSEA of ES and normal tissues based on KEGG pathways.

**Supplementary Table 3.** GSEA of ES and normal tissues based on Hallmark pathways.

**Supplementary Table 4.** Differentially expressed genes between ES and normal tissues in the combined cohort.

**Supplementary Table 5.** Differentially expressed genes between shHOXC6_1 and NC group.

**Supplementary Table 6.** Differentially expressed genes between shHOXC6_2 and NC group.

**Supplementary Table 7.** Downregulated gene sets.

**Supplementary Table 8.** GO enrichment analysis of downregulated gene sets.

**Supplementary Table 9.** KEGG enrichment analysis of downregulated gene sets.

**Supplementary Table 10.** Upregulated gene sets.

**Supplementary Table 11.** GO analysis of upregulated gene sets.

**Supplementary Table 12.** KEGG pathway analysis of upregulated gene sets.
