# Supplementary figures and images for: Identification and validation of HOXC6 as a diagnostic biomarker for Ewing sarcoma: insights from machine learning algorithms and in vitro experiments
Source: Front Immunol. 2025 Apr 4;16:1449355. doi: 10.3389/fimmu.2025.1449355 (PMC12006176; doi:10.3389/fimmu.2025.1449355)

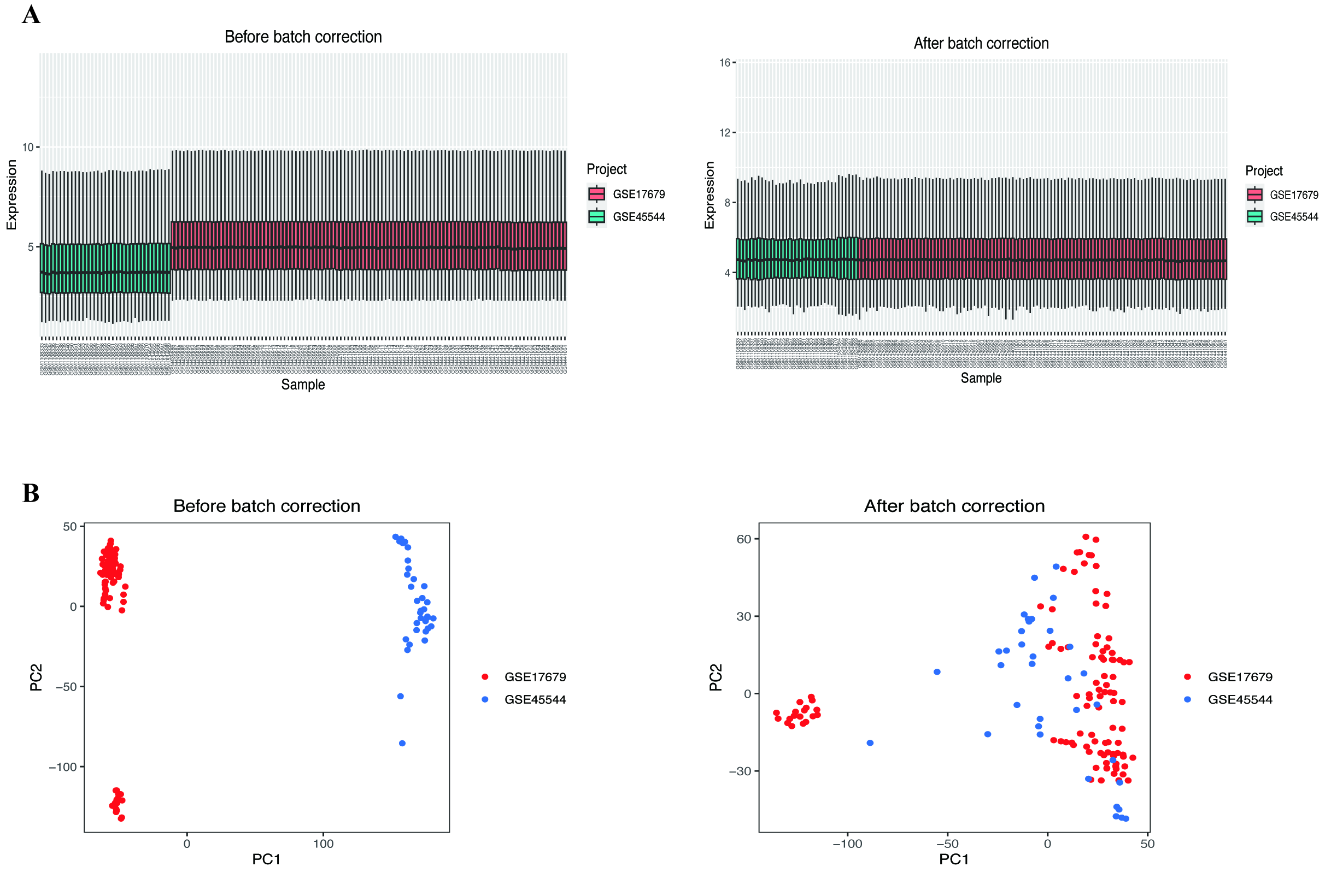

Supplement: Supplementary Table 1 — GSEA of ES and normal tissues based on GO biological processes. GSEA, gene set enrichment analysis; ES, Ewing sarcoma; GO, Gene Ontology. [file DataSheet2.zip › Supplementary material presentation/Supplementary Figure 1.tif]

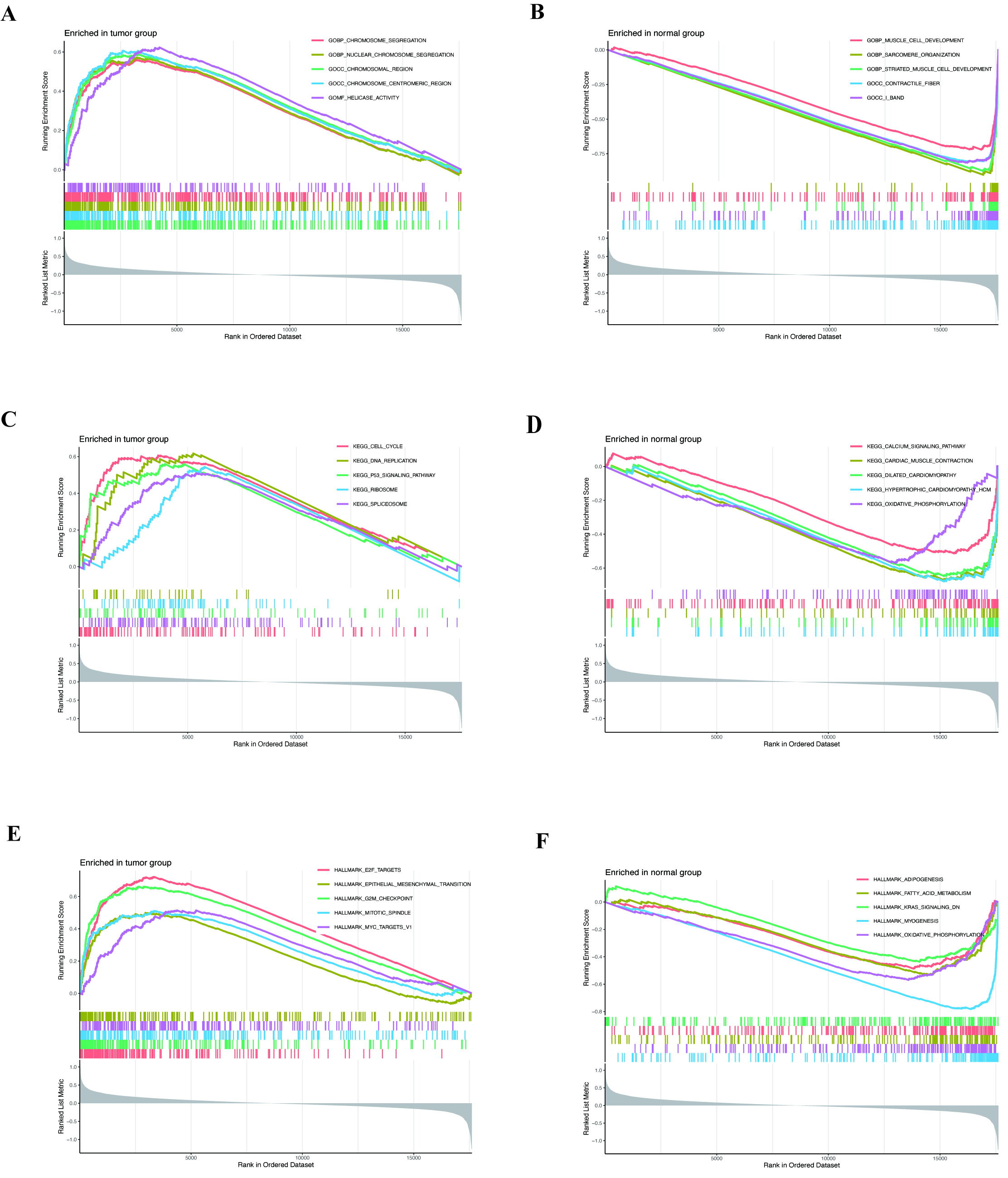

Supplement: Supplementary Table 1 — GSEA of ES and normal tissues based on GO biological processes. GSEA, gene set enrichment analysis; ES, Ewing sarcoma; GO, Gene Ontology. [file DataSheet2.zip › Supplementary material presentation/Supplementary Figure 2.tif]

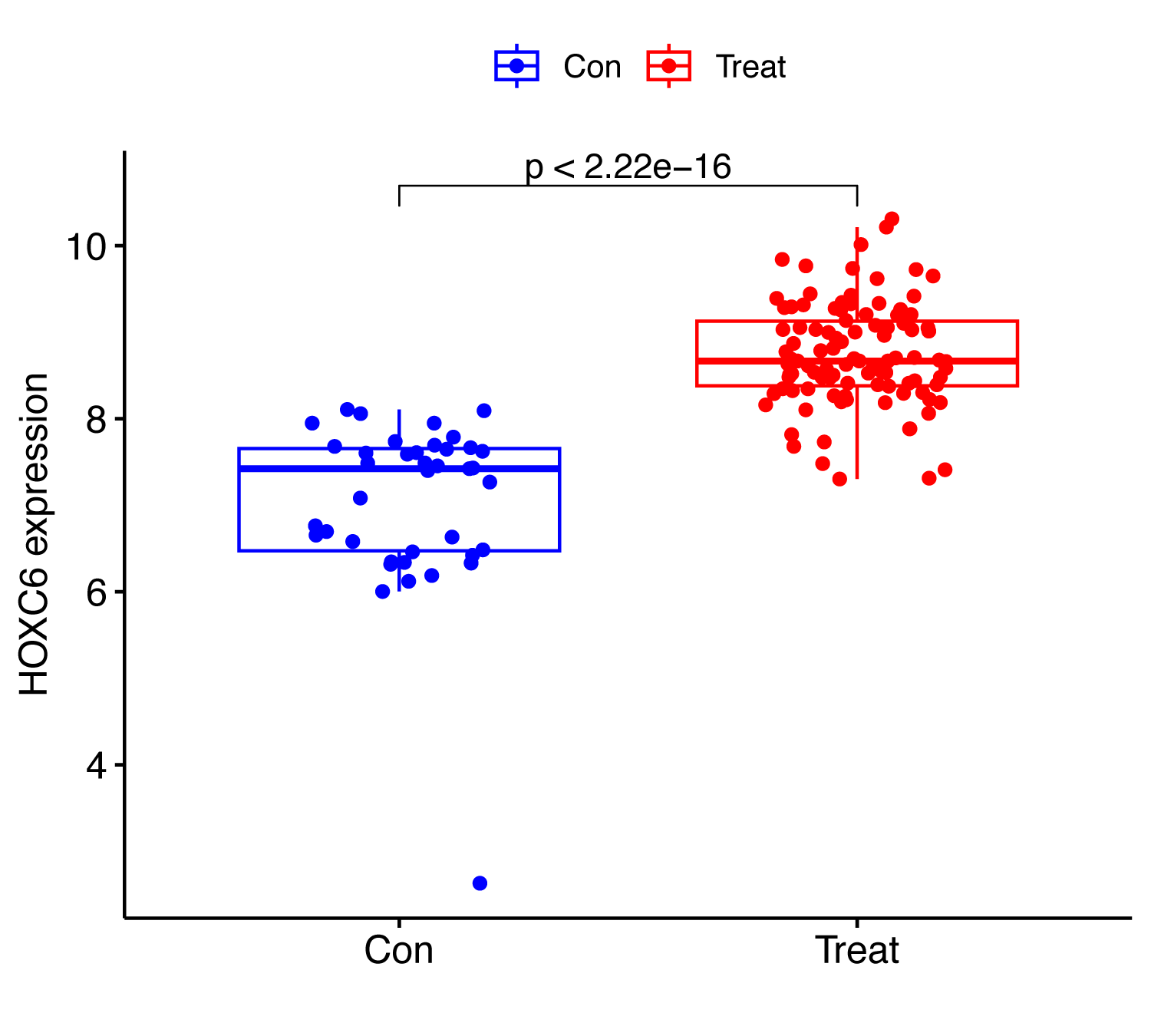

Supplement: Supplementary Table 1 — GSEA of ES and normal tissues based on GO biological processes. GSEA, gene set enrichment analysis; ES, Ewing sarcoma; GO, Gene Ontology. [file DataSheet2.zip › Supplementary material presentation/Supplementary Figure 3.tif]

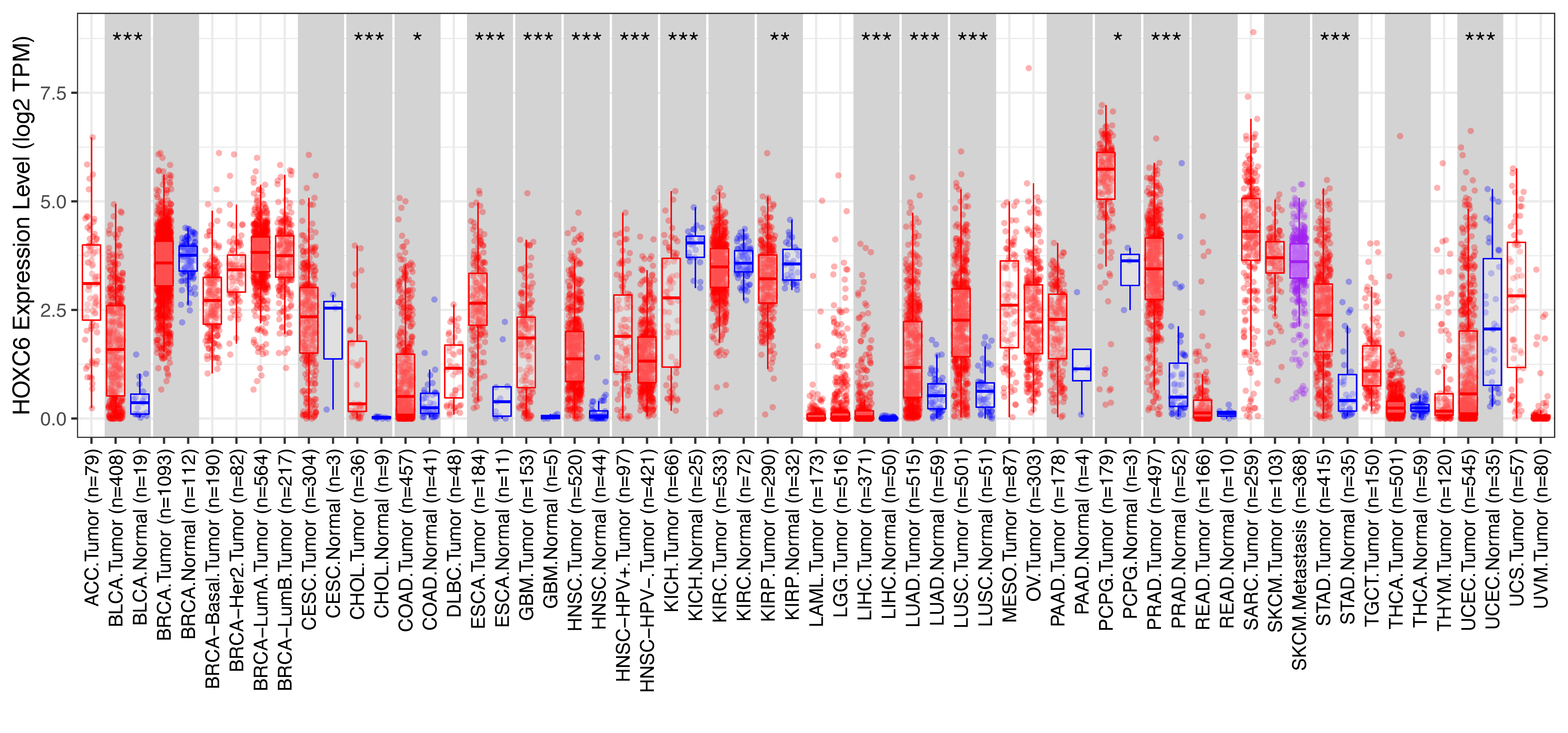

Supplement: Supplementary Table 1 — GSEA of ES and normal tissues based on GO biological processes. GSEA, gene set enrichment analysis; ES, Ewing sarcoma; GO, Gene Ontology. [file DataSheet2.zip › Supplementary material presentation/Supplementary Figure 4.tif]

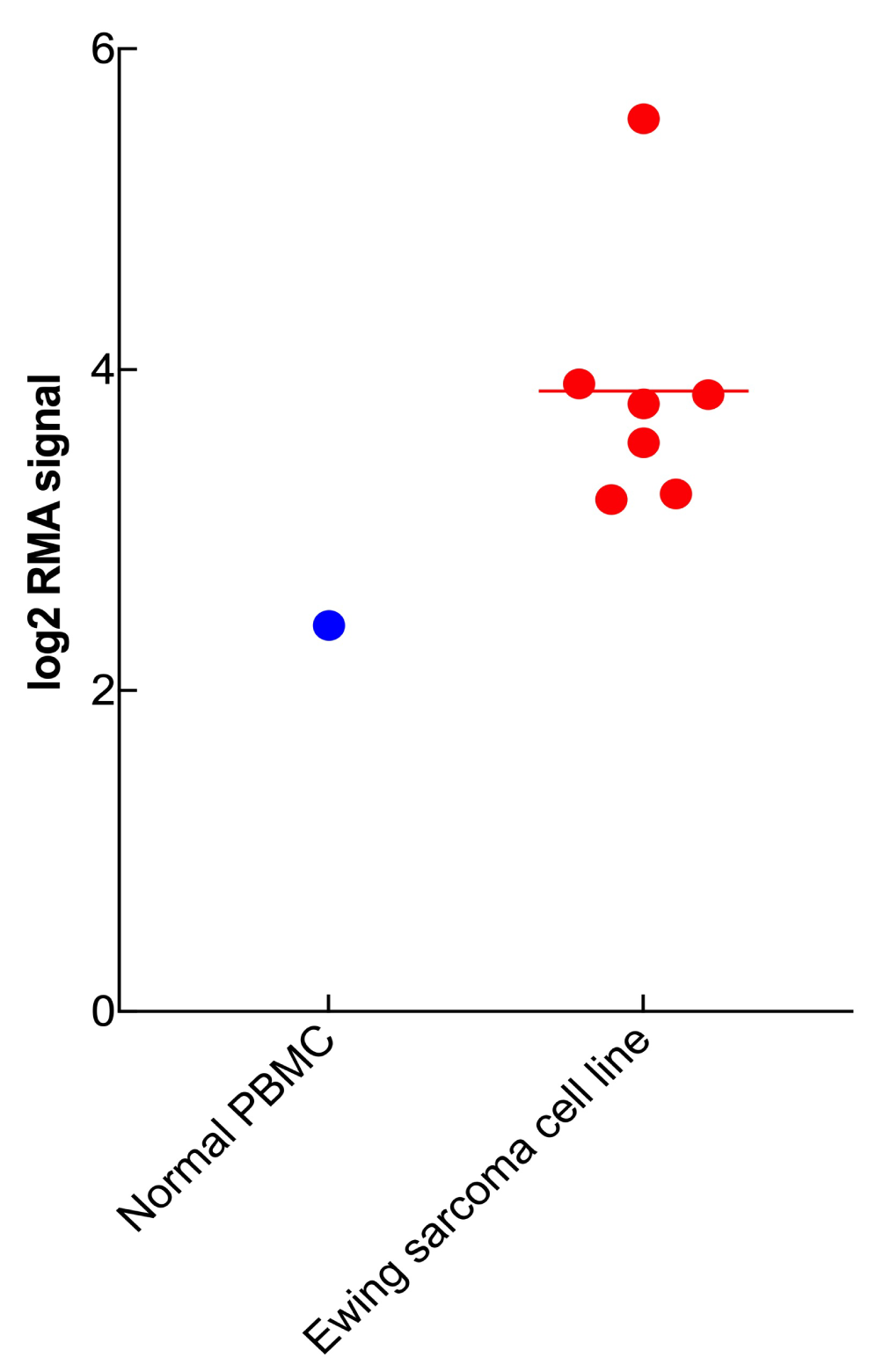

Supplement: Supplementary Table 1 — GSEA of ES and normal tissues based on GO biological processes. GSEA, gene set enrichment analysis; ES, Ewing sarcoma; GO, Gene Ontology. [file DataSheet2.zip › Supplementary material presentation/Supplementary Figure 5.tif]

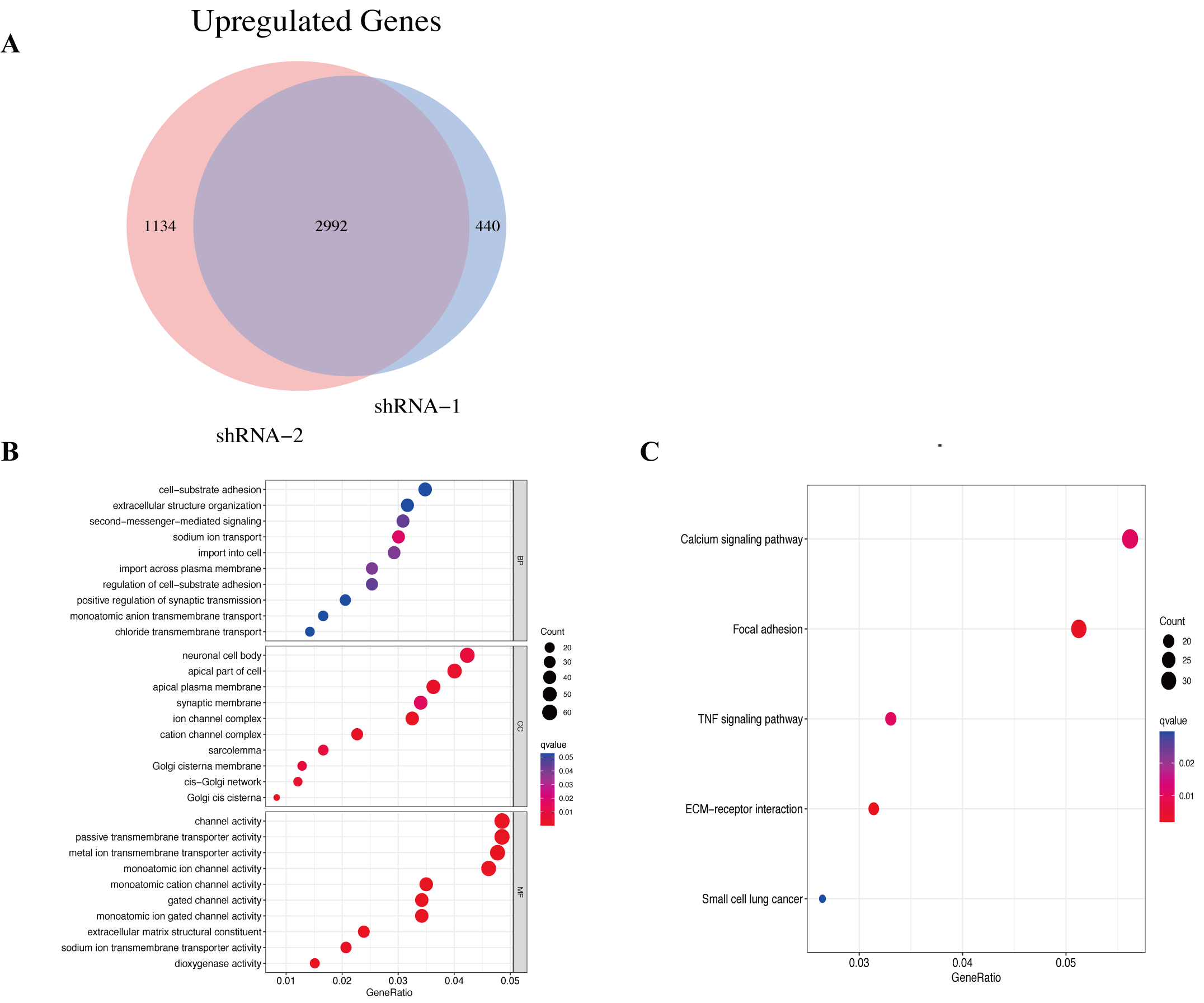

Supplement: Supplementary Table 1 — GSEA of ES and normal tissues based on GO biological processes. GSEA, gene set enrichment analysis; ES, Ewing sarcoma; GO, Gene Ontology. [file DataSheet2.zip › Supplementary material presentation/Supplementary Figure 6.tif]

Migration

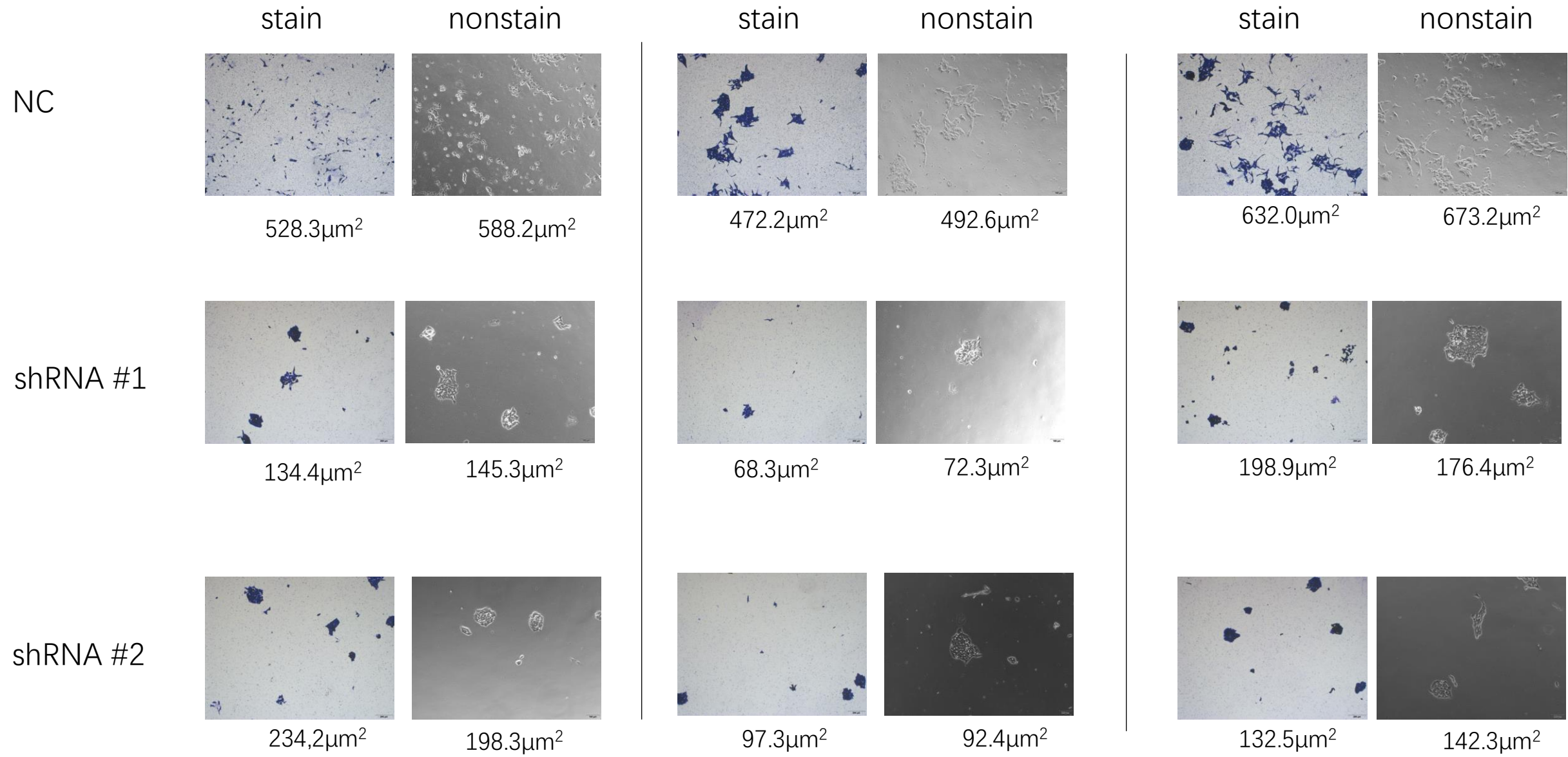

Supplement: Supplementary file 2 [file DataSheet1.zip › Transwell Assay.pdf]
