# Supplementary material for: Identification and validation of HOXC6 as a diagnostic biomarker for Ewing sarcoma: insights from machine learning algorithms and in vitro experiments
Source: Front Immunol. 2025 Apr 4;16:1449355. doi: 10.3389/fimmu.2025.1449355 (PMC12006176; doi:10.3389/fimmu.2025.1449355)

Repetation1

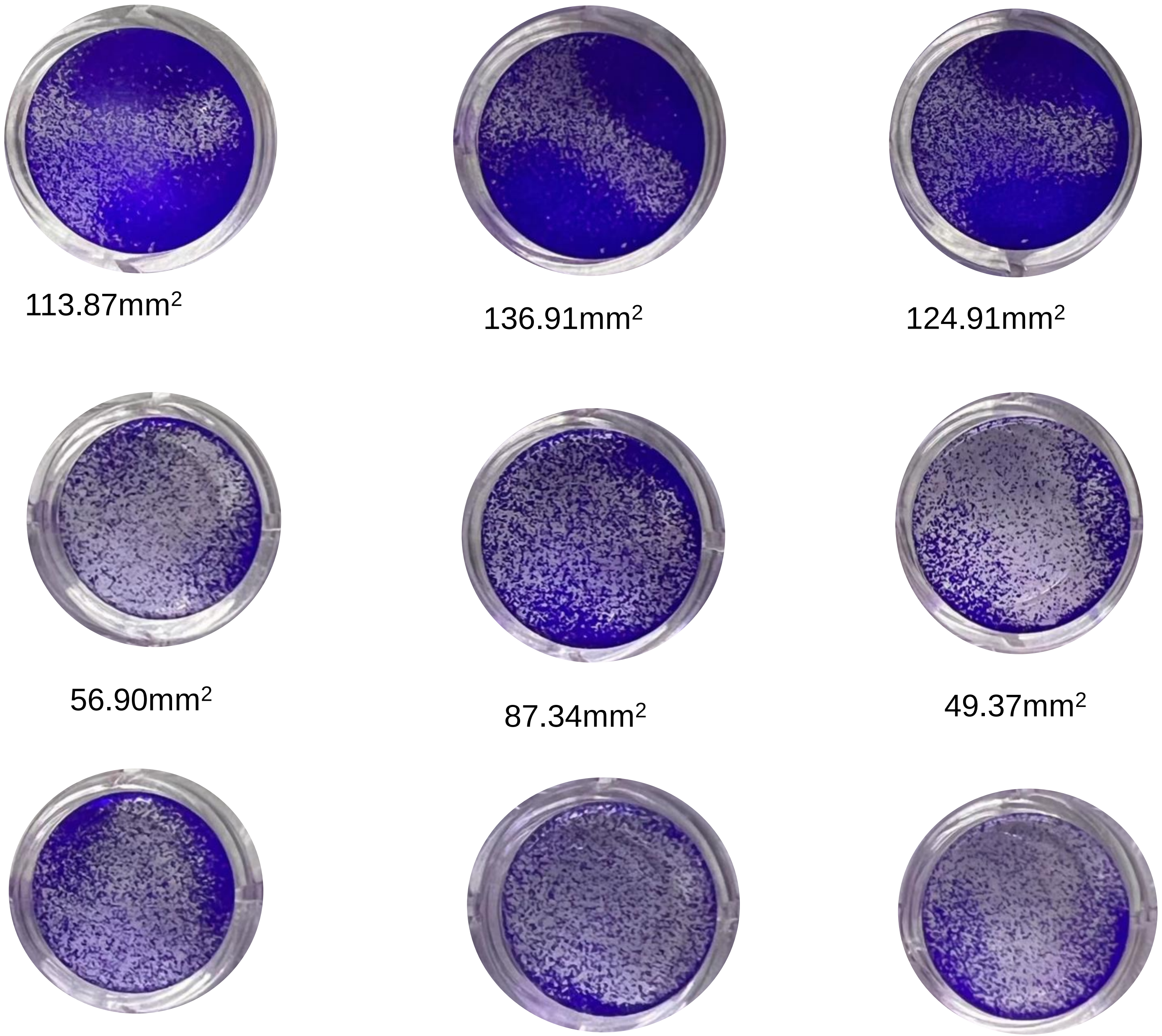

Repetation2

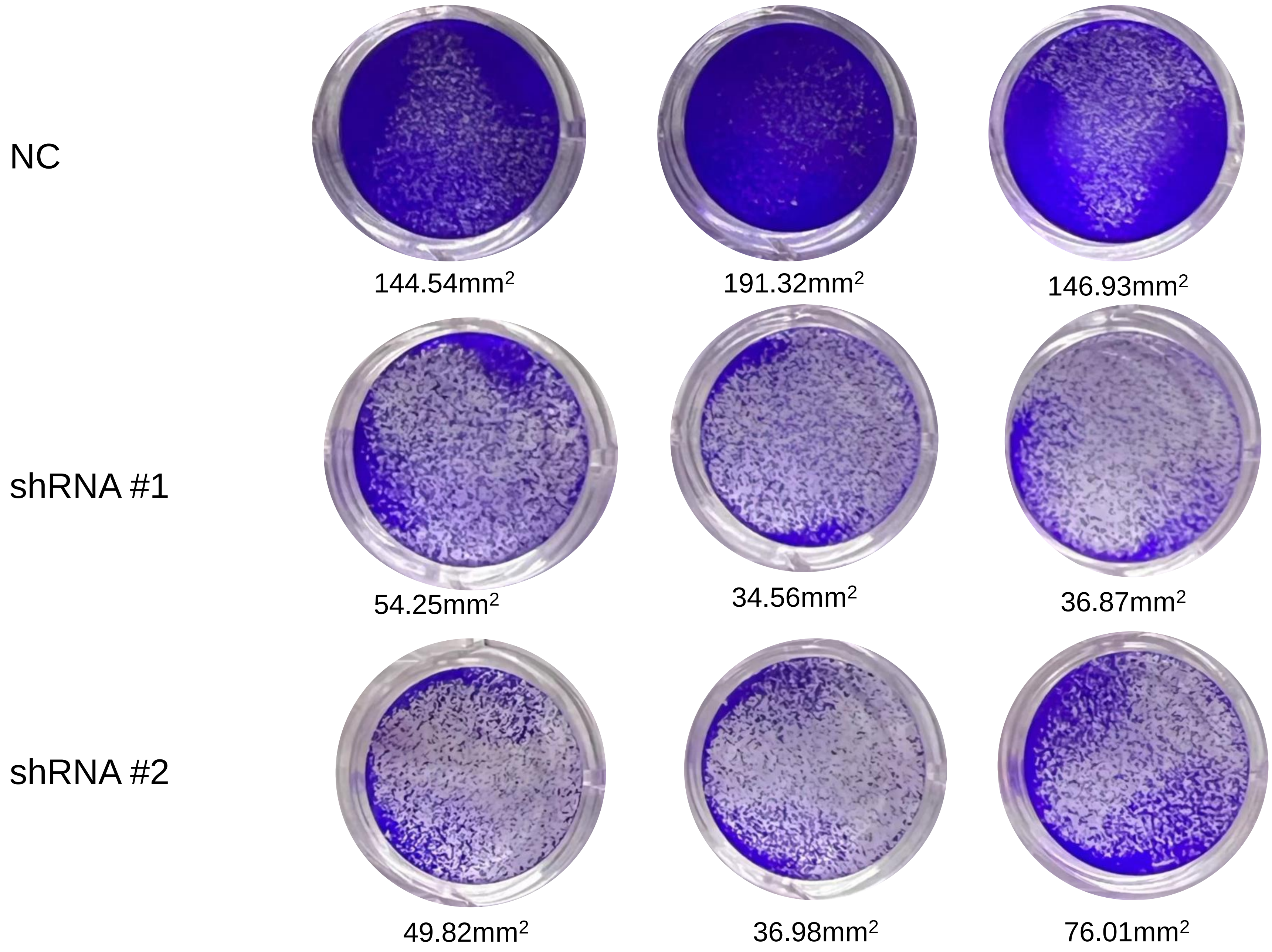

Repetation3

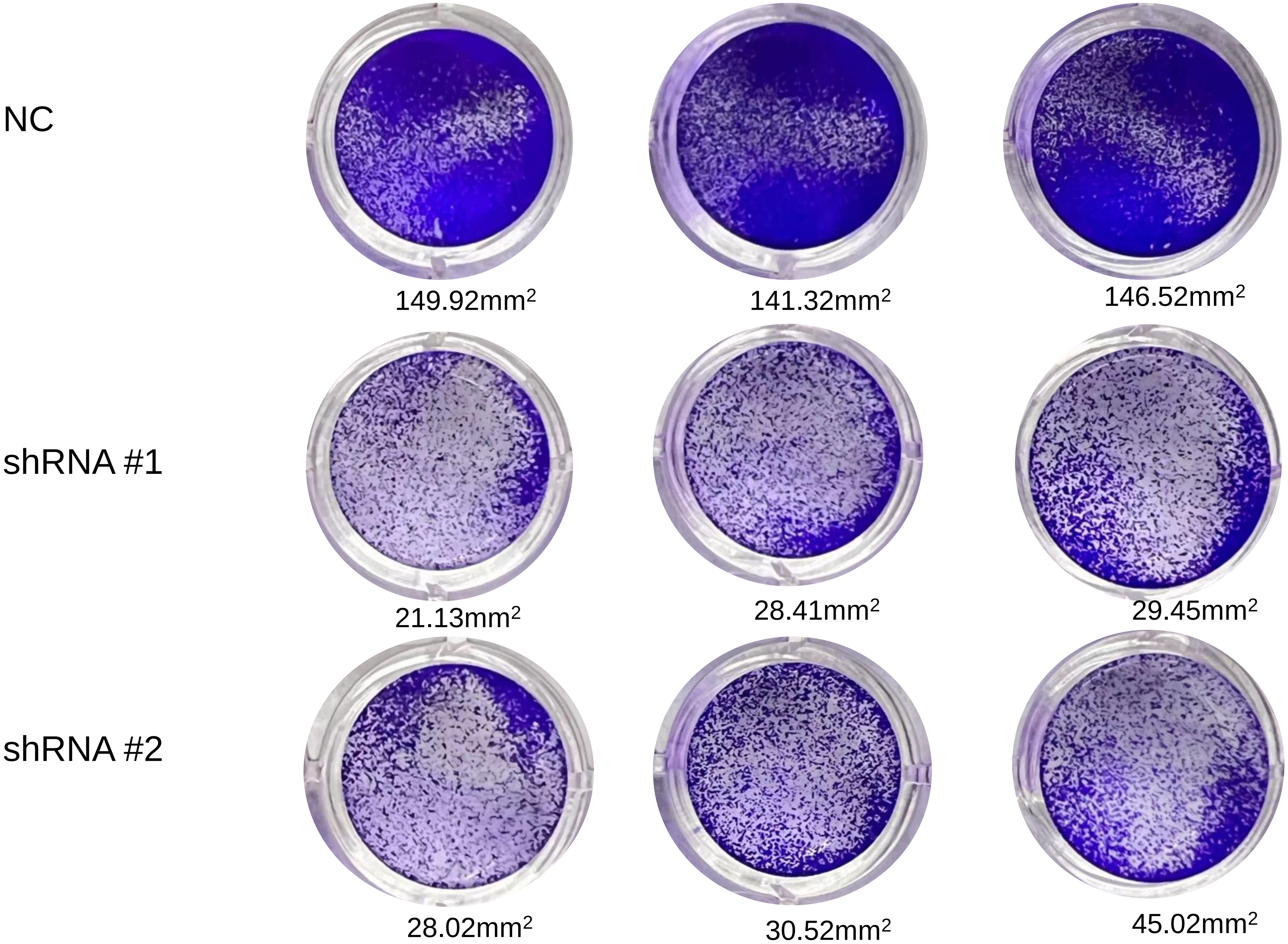

Repetation4

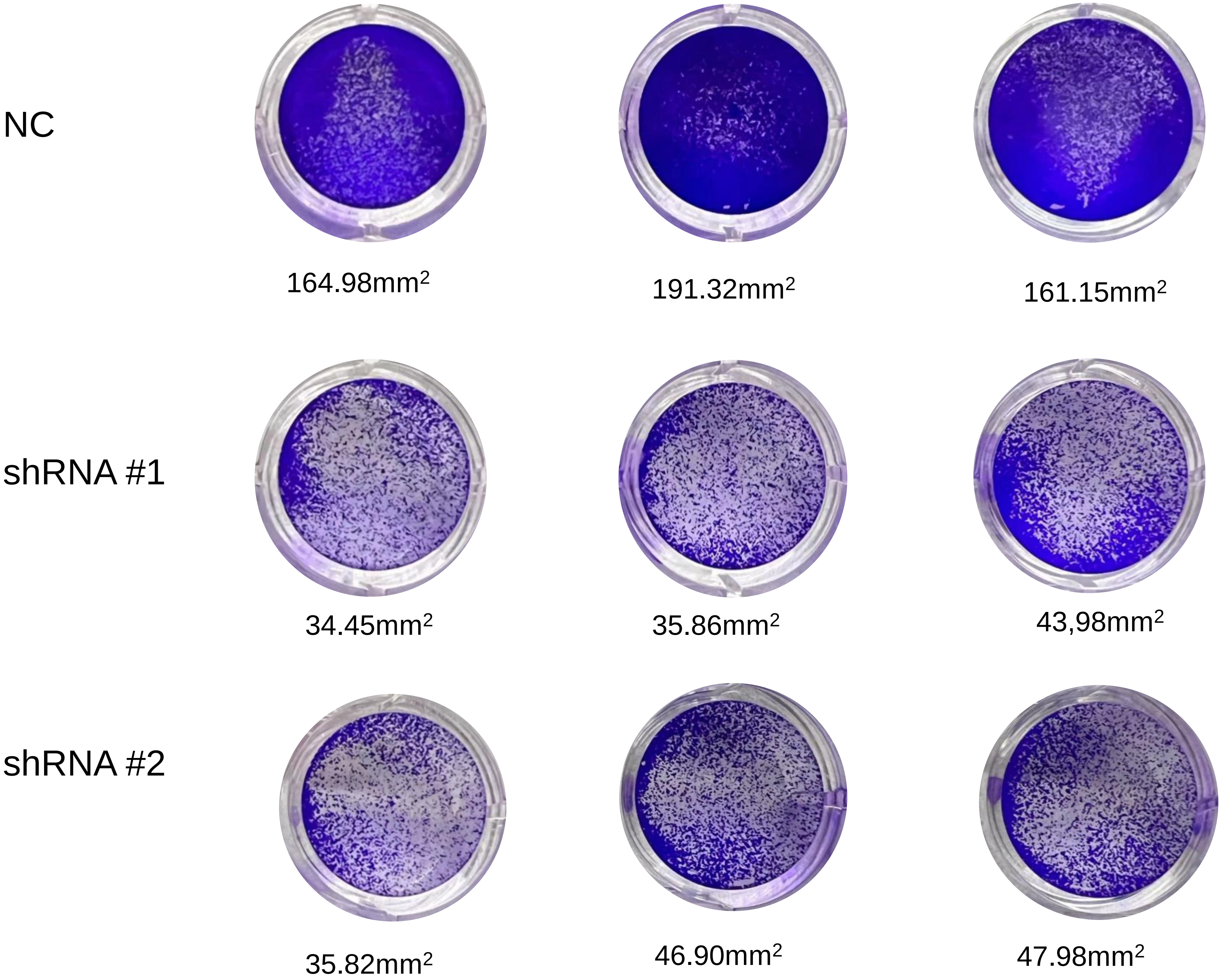

Supplement: Supplementary file 2 [file DataSheet1.zip › Colony Formation Assay.pdf]
