# Supplementary material for: Identification and validation of HOXC6 as a diagnostic biomarker for Ewing sarcoma: insights from machine learning algorithms and in vitro experiments
Source: Front Immunol. 2025 Apr 4;16:1449355. doi: 10.3389/fimmu.2025.1449355 (PMC12006176; doi:10.3389/fimmu.2025.1449355)

Wound healing assay 0h

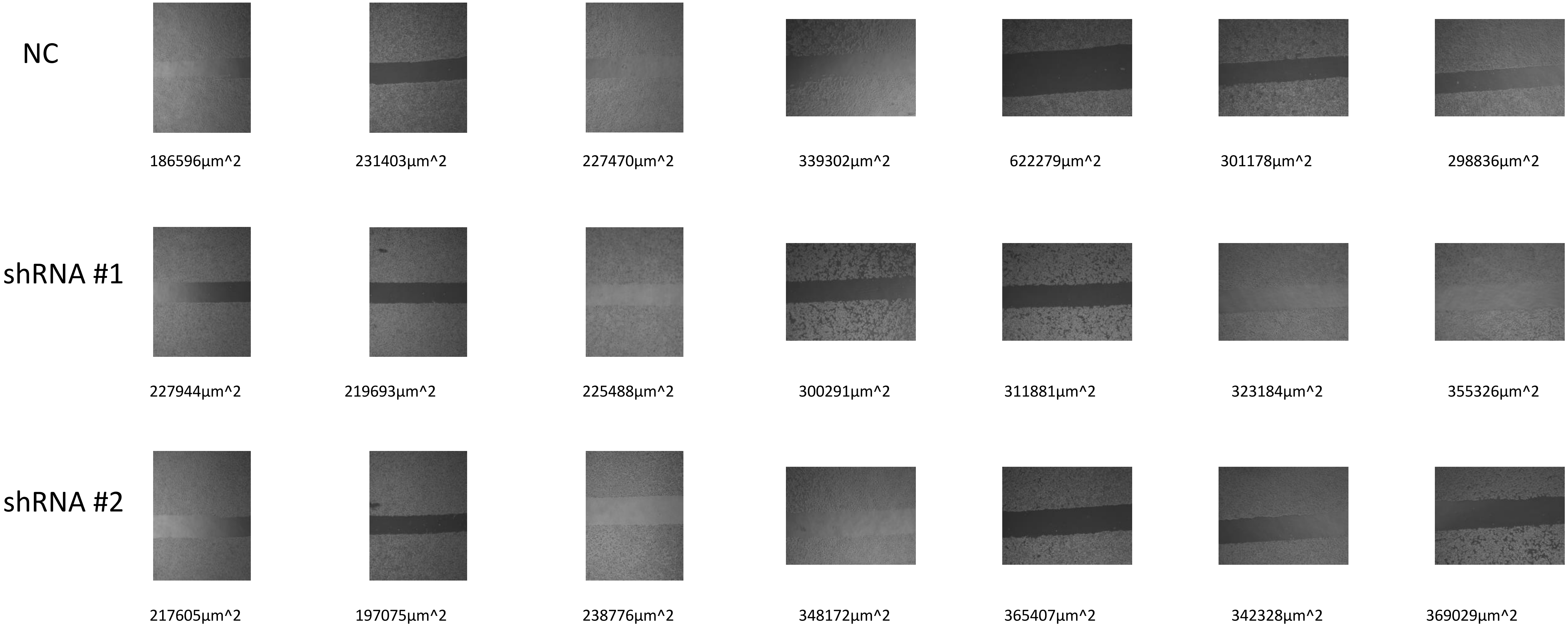

Blank area

Wound healing assay 24h

NC

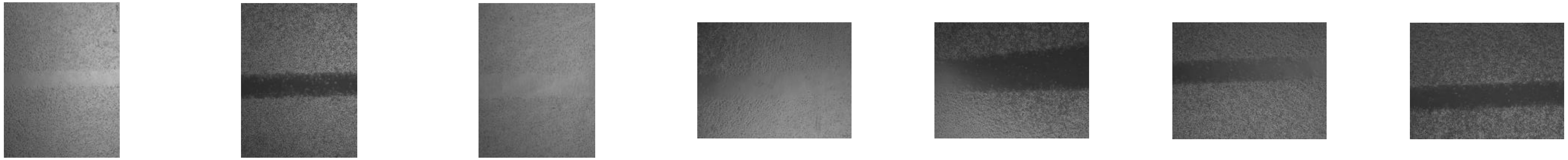

91275μm^2 108211μm^2 173108μm^2 276387μm^2 363678μm^2 180436μm^2 195737μm^2

shRNA #1

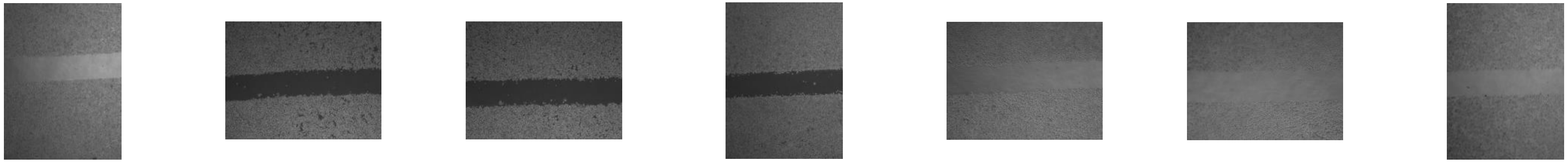

212830μm^2 217284μm^2 222494μm^2 287309μm^2 287309μm^2 300126μm^2 341052μm^2

shRNA #2

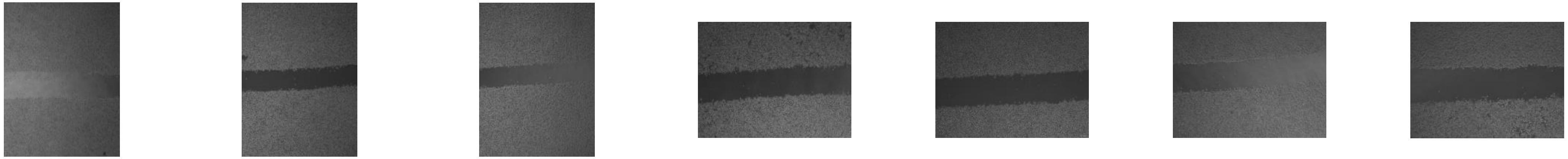

176355μm^2 176355μm^2 227041μm^2 328295μm^2 349766μm^2 314849μm^2 353590μm^2

Blank area

Wound healing assay 48h

NC

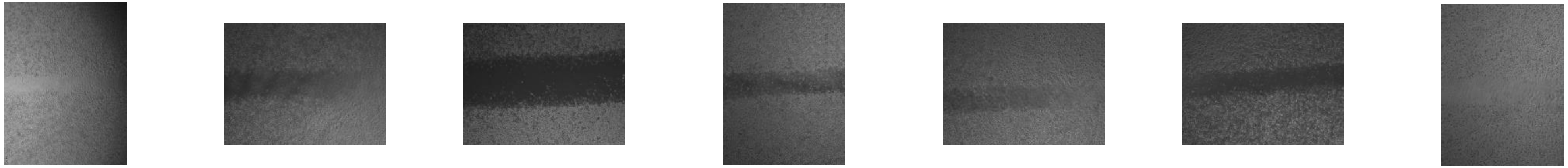

43890μm^2 | 32908μm^2 | 156736μm^2 | 200908μm^2 | 100091μm^2 | 150598μm^2 | 98717μm^2

shRNA #1

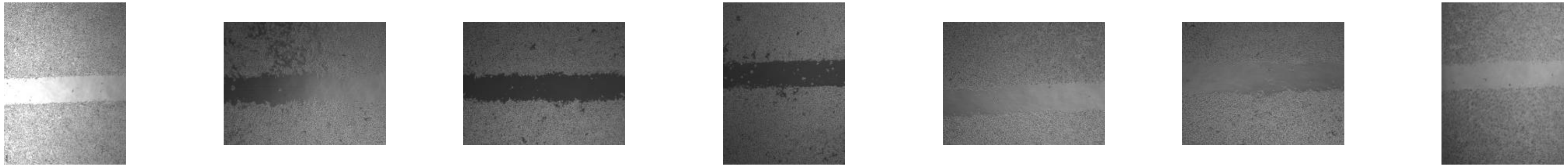

209672μm^2 | 212498μm^2 | 215678μm^2 | 264532μm^2 | 243490μm^2 | 283412μm^2 | 319098μm^2

shRNA #2

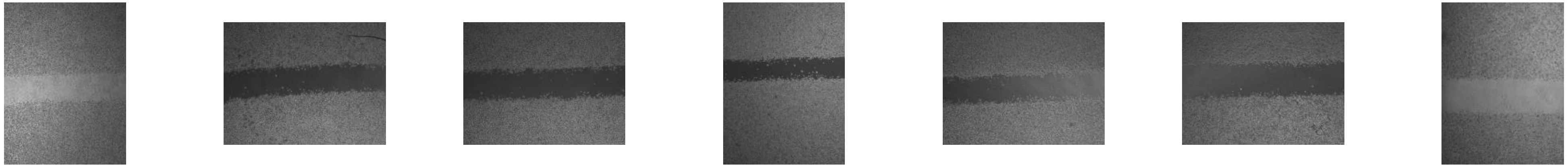

169980μm^2 | 167589μm^2 | 225065μm^2 | 324560μm^2 | 341010μm^2 | 308920μm^2 | 349098μm^2

Wound healing assay 72 h

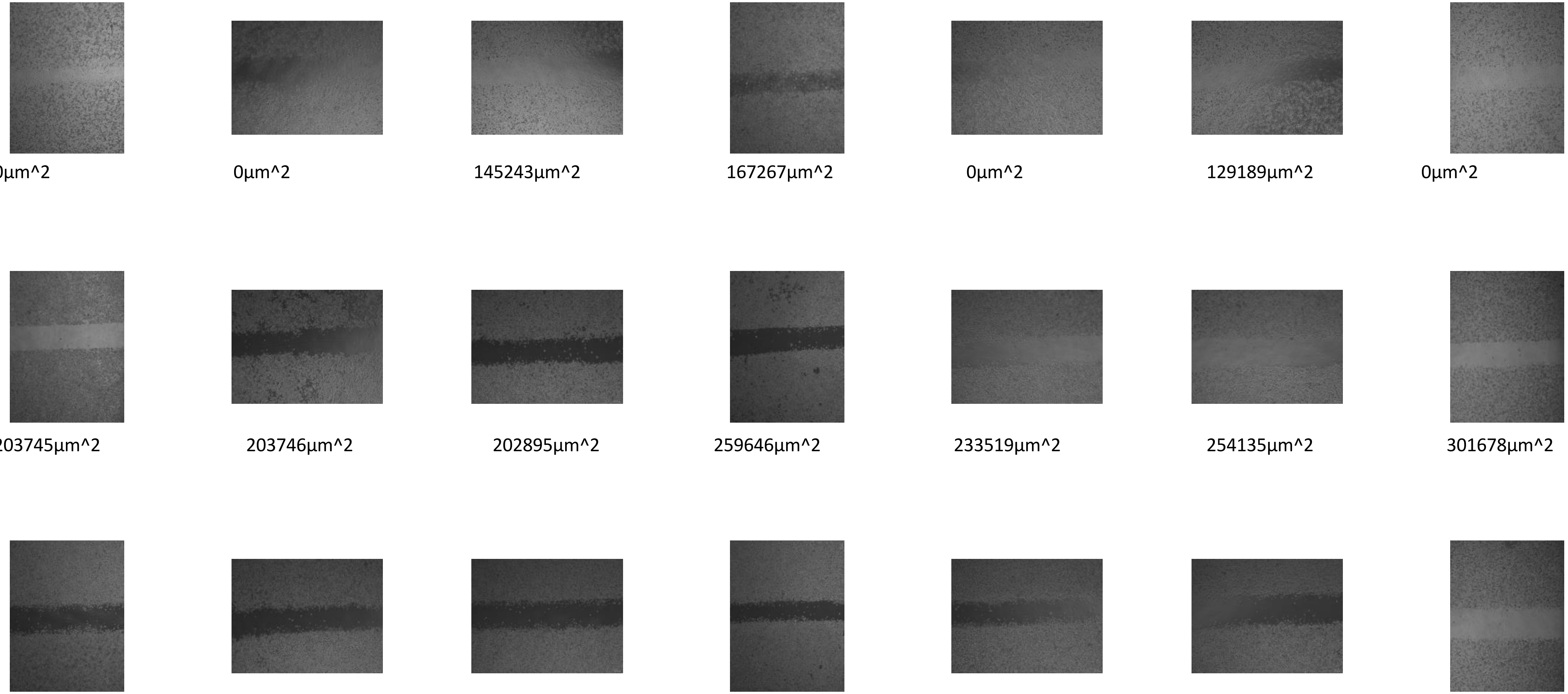

Supplement: Supplementary file 2 [file DataSheet1.zip › Wound Healing Assay.pdf]
